# Linking Protective *GAB2* Variants, Increased Cortical *GAB2* Expression and Decreased Alzheimer’s Disease Pathology

**DOI:** 10.1371/journal.pone.0064802

**Published:** 2013-05-28

**Authors:** Fanggeng Zou, Olivia Belbin, Minerva M. Carrasquillo, Oliver J. Culley, Talisha A. Hunter, Li Ma, Gina D. Bisceglio, Mariet Allen, Dennis W. Dickson, Neill R. Graff-Radford, Ronald C. Petersen, the Genetic and Environmental Risk for Alzheimer’s disease (GERAD1) Consortium, Kevin Morgan, Steven G. Younkin

**Affiliations:** 1 Department of Neuroscience, Mayo Clinic College of Medicine, Jacksonville, Florida, United States of America; 2 School of Molecular Medical Sciences, Queen’s Medical Centre, University of Nottingham, Nottingham, United Kingdom; 3 Department of Neurology, Mayo Clinic College of Medicine, Jacksonville, Florida, United States of America; 4 Department of Neurology and the Mayo Alzheimer Disease Research Center, Mayo Clinic College of Medicine, Rochester, Minnesota, United States of America; Emory University School Of Medicine, United States of America

## Abstract

GRB-associated binding protein 2 (*GAB2*) represents a compelling genome-wide association signal for late-onset Alzheimer’s disease (LOAD) with reported odds ratios (ORs) ranging from 0.75–0.85. We tested eight *GAB2* variants in four North American Caucasian case-control series (2,316 LOAD, 2,538 controls) for association with LOAD. Meta-analyses revealed ORs ranging from (0.61–1.20) with no significant association (all p>0.32). Four variants were hetergeneous across the populations (all p<0.02) due to a potentially inflated effect size (OR = 0.61–0.66) only observed in the smallest series (702 LOAD, 209 controls). Despite the lack of association in our series, the previously reported protective association for *GAB2* remained after meta-analyses of our data with all available previously published series (11,952-22,253 samples; OR = 0.82–0.88; all p<0.04). Using a freely available database of lymphoblastoid cell lines we found that protective *GAB2* variants were associated with increased *GAB2* expression (p = 9.5×10^−7^−9.3×10^−6^). We next measured *GAB2* mRNA levels in 249 brains and found that decreased neurofibrillary tangle (r = −0.34, p = 0.0006) and senile plaque counts (r = −0.32, p = 0.001) were both good predictors of increased *GAB2* mRNA levels albeit that sex (r = −0.28, p = 0.005) may have been a contributing factor. In summary, we hypothesise that *GAB2* variants that are protective against LOAD in some populations may act functionally to increase *GAB2* mRNA levels (in lymphoblastoid cells) and that increased *GAB2* mRNA levels are associated with significantly decreased LOAD pathology. These findings support the hypothesis that Gab2 may protect neurons against LOAD but due to significant population heterogeneity, it is still unclear whether this protection is detectable at the genetic level.

## Introduction

Genome-wide association studies (GWAS) represent an unbiased approach to identify susceptibility loci from complex diseases such as late-onset Alzheimer’s disease (LOAD). Besides the consistently reported *APOE* locus, several strong GWAS signals (eg. *BIN1, CLU*, *CR1* and *PICALM*) have recently been identified [1], [2], [3] and replicated in independent follow-up studies or GWAS [4], [5], [6], [7], [8], [9], [10]. As a result, variants at these loci currently (as of February 2013) show the strongest association with LOAD risk in meta-analyses of published studies for all LOAD candidates performed by the AlzGene forum, available at www.alzgene.org [11]. Historically, effect sizes and significance levels of many promising LOAD variants have diminished following publication of multiple independent case-control association studies, thus highlighting the importance of more follow-up studies for these putative variants.

The genetic locus encoding *GRB-associated binding protein 2* (*GAB2*) is an example of a candidate gene that has shown relatively consistent replication in eleven published studies since its identification in 2007 [12] and still remains a strong LOAD candidate [13]. The *GAB2* signal was initially identified as a LOAD candidate in *APOE* ε3ε4 carrier and non-carrier subgroups of a LOAD GWAS [12]; within the discovery subgroup of *APOE* ε3ε4 carriers, 10 of the 25 most significant variants associated with LOAD were located in *GAB2* on chromosome 11q14.1. Combining data from neuropathological and clinical replication cohorts revealed highly significant associations of all ten *GAB2* variants (9.7×10^−11^<p<1.2×10^−5^) with LOAD. The numerous follow-up case-control association studies of *GAB2* and subsequent GWAS have provided further support for *GAB2* as a strong candidate LOAD gene; while only four [14], [15], [16], [17] of the nine studies of Caucasian European populations [1], [5], [14], [15], [16], [17], [18], [19], [20] successfully replicated the association observed by Reiman *et al*., individually, meta-analyses of all published studies performed by AlzGene [11] reported significant ORs for all ten *GAB2* variants in the Caucasian studies (most studied variant: rs4945261, OR = 0.79, 95%CI 0.66–0.94). Although the *GAB2* variants were not significantly associated with LOAD in Japanese [21] or Han Chinese populations [22], addition of these data to the meta-analysis still revealed a significant pooled odds ratio for all variants (most studied variant: rs4945261, OR = 0.82, 95%CI 0.70–0.95). Overall, these studies provide good genetic evidence for *GAB2* as a LOAD candidate worthy of further investigation.

Here we have genotyped eight *GAB2* variants identified by Reiman *et al* in our large, case-control association series (2,316 LOAD and 2,538 controls) in an attempt to replicate and further strengthen the genetic association of *GAB2* with LOAD. In our previous publication [23], which included case-control association of the *GAB2* variant, rs10793294, we observed significant population heterogeneity of *GAB2* between our case-control series (p = 0.0002) and between all published series (p<0.0001). Despite this heterogeneity, meta-analysis of all published studies revealed significant association of rs10793294 with LOAD (minor allele OR = 0.74, p = 0.007). These findings suggested that either there was population-specific association of this *GAB2* variant such that the association in some populations is strong enough to withstand the dilution caused when the data are combined with populations that show no significant association or that some populations lacked the necessary statistical power to detect the significant association. To examine this further we provide statistical tests for population heterogeneity for all eight variants and meta-analyses of all available published data for these eight variants analyzing a total of 11,952-22,253 samples. We have also tested for association of *GAB2* haplotypes with *GAB2* mRNA levels using a) a database made available by Dixon et al [24] and b) post-mortem cerebellum and temporal cortex samples. Finally, we have tested the *GAB2* mRNA levels in temporal cortex and cerebellum for association with senile plaque and neurofibrillary tangle counts. This study therefore represents a thorough investigation of *GAB2* at the genetic, transcript and pathological level.

## Methods

### Ethics Statement

Approval was obtained from the ethics committee or institutional review board of each institution responsible for the ascertainment and collection of samples (Mayo Clinic College of Medicine, Jacksonville, FL and Mayo Clinic College of Medicine, Rochester, MN, USA, National Cell Repository for Alzheimer’s disease, Indianapolis. IN, USA). Written informed consent was obtained for all individuals that participated in this study.

### Case-control subjects

The case-control series consisted of 4,968 Caucasian subjects from the United States (2,316 LOAD, 2,538 control) ascertained at the Mayo Clinic (1,728 LOAD, 2,329 controls) or through the National Cell Repository for Alzheimer’s Disease (NCRAD: 588 LOAD, 209 control). All subjects ascertained at the Mayo Clinic in Jacksonville, Florida (JS: 589 LOAD, 593 control) and at the Mayo Clinic in Rochester, Minnesota (RS: 553 LOAD, 1,374 control) were diagnosed by a Mayo Clinic neurologist. The neurologist confirmed a Clinical Dementia Rating score of 0 for all JS and RS subjects enrolled as controls; cases had diagnoses of possible or probable LOAD made according to NINCDS-ADRDA criteria [25]. In the autopsy-confirmed series (AUT: 586 LOAD, 362 control) all brains were evaluated by Dr. Dennis Dickson and came from the brain bank maintained at the Mayo Clinic in Jacksonville. The diagnosis of definite AD was made according to NINCDS-ADRDA criteria. All LOAD brains analyzed in the study had a Braak score of 4.0 or greater. Brains employed as controls had a Braak score of 2.5 or lower but often had brain pathology unrelated to AD and pathological diagnoses that included vascular dementia, fronto-temporal dementia, dementia with Lewy bodies, corticobasal degeneration, argyrophilic grain disease, multi-system atrophy, amyotrophic lateral sclerosis, and progressive supra-nuclear palsy. One LOAD case from each of the 588 late-onset NCRAD families was analyzed. NCRAD LOAD cases were selected based on strength of diagnosis (autopsy-confirmed: 38%> probable: 54%> possible: 10%); the case with the earliest age at diagnosis was taken when several cases had equally strong diagnoses. The 209 NCRAD controls that we employed were unrelated Caucasian subjects from the United States with a Clinical Dementia Rating of 0, specifically collected for inclusion in case-control series. The number of *APOE* ε4+ and ε4- individuals, females and mean age at diagnosis/entry in the LOAD cases and controls for each series are shown in Table 1. In the meta-analyses of all published data, genotype counts were requested from Harold et al. [1]. These samples were provided by the Genetic and Environmental Risk for Alzheimer’s disease (GERAD1) Consortium, comprising 3333 cases and 1225 elderly screened controls genotyped at the Sanger Institute on the Illumina 610-quad chip. In this study, we included the UK samples (recruited by the Medical Research Council (MRC) Genetic Resource for AD (Cardiff University; Kings College London; Cambridge University; Trinity College Dublin), the Alzheimer’s Research Trust (ART) Collaboration (University of Nottingham; University of Manchester; University of Southampton; University of Bristol; Queen’s University Belfast; the Oxford Project to Investigate Memory and Ageing (OPTIMA), Oxford University); MRC Prion Unit, University College London; London and the South East Region AD project (LASER-AD), University College London; Competence Network of Dementia (CND) and the German samples (recruited by Department of Psychiatry, University of Bonn, Germany). The US samples from the Mayo Clinic were excluded here due to overlap with our samples. All AD cases met criteria for either probable (NINCDS-ADRDA, DSM-IV) or definite (CERAD) AD. All elderly controls were screened for dementia using the MMSE or ADAS-cog, were determined to be free from dementia at neuropathological examination or had a Braak score of 2.5 or lower.

**Table 1:** Details of samples used in this study.

	N			E4+; N (freq)		E4-; N (freq)		Females; N(freq)			Mean Age (range)	
Series	LOAD	CTRL	Total	LOAD	CTRL	LOAD	CTRL	LOAD	CTRL		LOAD	CTRL
JS	589	593	1,182	367 (0.62)	152 (0.26)	222 (0.38)	441 (0.74)	366 (0.62)	357 (0.60)		78.2 (61-95)	77.9 (60-100)
RS	553	1,374	1,927	309 (0.56)	340 (0.25)	244 (0.48)	1,034 (0.75)	346 (0.63)	747 (0.54)		79.6 (61-104)	78.4 (60-99)
AUT	586	362	948	363 (0.62)	80 (0.22)	223 (0.38)	282 (0.78)	345 (0.59)	154 (0.43)		81.1 (61-105)	75.8 (61-98)
Subtotal	1,728	2,329	4,057	1,039 (0.60)	572 (0.25)	689 (0.40)	1,757 (0.75)	1,057 (0.61)	1,258 (0.54)		79.6 (61-105)	77.9 (60-100)
NCRAD	588	209	797	467 (0.79)	34 (0.16)	121 (0.21)	175 (0.84)	398 (0.68)		129 (0.62)	75.3 (61-98)	78.3 (61-99)
**Total**	**2,316**	**2,538**	**4,854**	**1,498 (0.65)**	**606 (0.24)**	**818 (0.35)**	**1,932 (0.76)**	**1,455 (0.63)**		**1,387 (0.55)**	**78.6 (61-105)**	**77.9 (60-100)**

**B)** Variant ID, base pair position (BP) on Chromosome 11 (genomic contig reference assembly), genotype counts (11 = major allele homozygote, 12 = heterozygote, 22 = minor allele homozygote) and minor allele frequencies (MAF) are shown for each case-control series and in the total dataset.

The number of LOAD patients (AD) and controls (CTRL), APOE ε4 carriers (E4+) and non-carriers (E4–), females and mean age (at diagnosis/entry) are given for each individual Mayo Clinic series, the Mayo Clinic subtotal, the NCRAD series and the total dataset.

### DNA isolation

For the JS and RS samples, DNA was isolated from whole blood using an AutoGen instrument (AutoGen, Inc, Holliston, MA). The DNA from AUT samples was extracted from cerebellum using Wizard® Genomic DNA Purification Kits (Promega Corp., Madison, WI). DNA from the RS and AUT series was scarce, so samples from these two series were subjected to whole genome amplification using the Illustra GenomiPhi V2 DNA Amplification Kit (GE Healthcare Bio-Sciences Corp., Piscataway, NJ).

### Genotyping of variants

We genotyped eight of the ten *GAB2* variants included in the Reiman *et al.* study; rs2510038 was not genotyped since it was not compatible with our Sequenom genotyping multiplex PCR pool and rs10793294 was not included in the single variant association part of this study since we have previously published significant association of this variant with LOAD [23] but was included in the latter part of the study. Genotyping of rs10793294 is described in our previous publication. The remaining eight variants were genotyped using SEQUENOM’s MassArray iPLEX technology (SEQUENOM Inc, San Diego, CA) following the manufacturer’s instructions. Genotype calls were made using the default post-processing calling parameters in SEQUENOM Typer 4.0 software, followed by visual inspection to remove genotype calls that were obviously erroneous, based on the presence or absence of allele peaks in an individual sample spectrogram.

### Measurement of *GAB2* mRNA Expression

Total RNA was extracted from 356 samples of cerebellum and 163 samples of temporal cortex from LOAD brains and controls using an ABI PRISM 6100 Nucleic Acid PrepStation and the Total RNA Isolation Chemistry kit from Applied Biosystems. RNA was reverse transcribed to single-stranded cDNA using the High-Capacity cDNA Archive Kit from Applied Biosystems. Real-time quantitative PCR was performed in triplicate for each sample using ABI TaqMan Low Density expression Arrays (384-Well Micro Fluidic Cards) with a pre-validated TaqMan Gene Expression Assay. 18s ribosomal RNA (18s rRNA) was used as the endogenous control for the relative quantification of *GAB2* mRNA. Real-time PCR cycle threshold (C_T_) raw data was collected and exported using the ABI PRISMH SDS software version 2.2. The variable C_T_ within the raw data file indicates the PCR cycle number at which the amount of amplified gene target reaches a fixed threshold. The variable ΔC_T_ denotes the difference between the averaged C_T_ values for the *GAB2* transcript and that for the reference 18S rRNA transcript. The ΔC_T_ values calculated from each sample were used as quantitative phenotypes to determine associations between *GAB2* haplotypes and the level of *GAB2* transcript. Some samples had one or more replicate measurements that failed to amplify and were obvious outliers, thus they were excluded from the analysis.

### Pathological measures

Of the postmortem samples, neuropathological data was available for 128 LOAD patients and 121 controls. Senile plaques and neurofibrillary tangles were counted in three cortical sections with thioflavin-S fluorescent microscopy. All neuropathological assessments were blinded to the medical records. All aspects of the study were approved by the Mayo Institutional Review Board.

### Statistical Analyses

**Single variant case-control association study:** Breslow-Day tests and meta-analyses were performed using StatsDirect v2.5.8 software. Summary ORs and 95% CI were calculated using the Dersimonian and Laird (1986) random-effects model. Genotype counts for published data were taken from the AlzGene website. In the case of the Reiman et al. data, genotype counts from the total dataset (and not from the *APOE* E4+ or E4– subgroups) were used. In the case of the Logistic regression (allelic model) correcting for *APOE ε*4 dose (0, 1 or 2 copies of the *APOE ε*4 allele), sex and age-at-diagnosis were performed using StatsDirect v2.5.8 software. **Haplotype estimation and association study:** Haplotype frequencies were estimated using the expectation-maximization approach implemented in the haplo.em function of Haplo.stats v1.2.2 [26] using R programming software. Global haplotype association and individual haplotype score tests corrected for *APOE ε*4 dose, sex and age-at-diagnosis were performed using the haplo.score function of Haplo.stats v1.2.2. **Conserved region search:** A search was performed for >70% identity (the default parameter for defining a conserved element [27]) over 100 bp windows between the human (Human April 2003 genome build) and mouse (February 2003 build) sequence as determined by the pre-computed alignments in the VISTA Genome Browser (http://pipeline.lbl.gov/cgi-bin/gateway2). **Epistatic Interaction:** All nine *GAB2* variants and the two variants in APOE that confer allelic status (rs7412 and rs429358) were tested for pair-wise epistatic interaction using the –epistasis function of PLINK v1.07 (http://pngu.mgh.harvard.edu/purcell/plink/) [28]. Covariates could not be included in these analyses due to software limitations. **Association of** ***GAB2*** **mRNA levels (ΔC_T_) with covariates and pathological traits:** Spearman correlations, chi-squared and independent t-tests were performed in StatsDirect v2.5.8 software. Age-at-death, neurofibrillary tangle counts and senile plaque counts were included as continuous traits, while *APOE ε*4 dose (0, 1, 2) and sex were included as categorical traits.

## Results

### No association of *GAB2* variants with LOAD risk in 2,316 LOAD patients and 2,538 controls from North America

**Figure 1:**
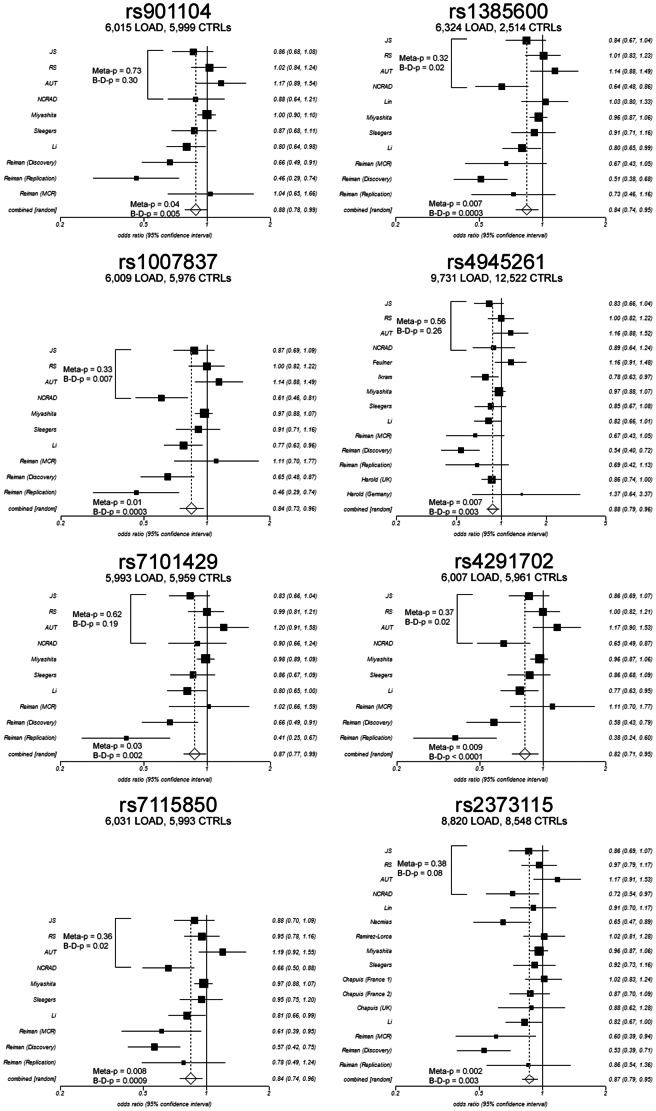
Meta-analyses of each variant in our four case-control series and all series published to-date. The total number of LOAD and controls used for each meta-analysis and Breslow-Day (B–D) p-value for all series are shown at the top of each graph. ORs (boxes) and 95%CI (whiskers) are plotted for each population and tabulated on the right of each graph. Combined OR is the overall OR calculated by the meta-analysis using a random effects model. Meta-analysis p-value for all series is shown to the left of the combined odds ratio. Breslow-Day and Meta-analysis p-values for our data only are shown next to the OR plots for JS, RS, AUT and NCRAD series.

We genotyped eight *GAB2* variants in our large case-control dataset (Table 1) that includes 2,316 LOAD patients and 2,538 controls of North American Caucasian descent. Table 2 shows the genotype counts for these eight variants in each series. All variants were in Hardy-Weinberg equilibrium (all p>0.1). As shown in Figure 1, meta-analyses of our four case-control series (JS, RS, AUT, NCRAD) revealed no association overall for any of the eight variants with LOAD risk (all Meta p>0.3) albeit that five variants were associated with LOAD in the NCRAD series; rs1385600 (OR = 0.64, 95%CI 0.48–0.86), rs1007837 (OR = 0.61, 95%CI 0.46–1.81), rs4291702 (OR = 0.65, 95%CI 0.49–0.87), rs7115850 (OR = 0.66, 95%CI 0.50–0.88) and rs2373115 (OR = 0.72, 95%CI 0.54–0.97). Population heterogeneity for the four variants (Breslow-Day p-values; rs1385600 p = 0.01, rs1007837 p = 0.006, rs4291702 p = 0.02, rs7115850 p = 0.02) disappeared when the NCRAD series was removed from the analysis (all p>0.11; data not shown). It must be noted that NCRAD was the smallest of the four case-control series, suggesting the possibility that the increased frequency observed in the 209 controls could in fact be an artefact. Nevertheless, the protective associations for the minor allele of these five variants in the NCRAD series successfully replicate the risk associations reported for the major alleles of the same variants reported in the Reiman *et al* study. In order to further evaluate the association in the NCRAD series and to determine whether they were independent of covariates, we performed logistic regression for all eight variants correcting for *APOE* ε4 dose (0, 1 or 2 copies of the *APOE* ε4 allele), sex and age-at-diagnosis/sampling in the NCRAD series (Table 3). Effect sizes for these five associations did not remain following adjustment for covariates (all p>0.09) indicating that the associations were not independent associations.

**Table 2:** Genotype counts for each case-control series.

		JS LOAD (589)				JS CTRL (593)				RS LOAD (553)				RS CTRL (1374)				AUT LOAD (586)				AUT CTRL (362)				NCRAD LOAD (588)				NCRAD CTRL (n = 209)			
Variant ID	Alleles	11	12	22	MAF	11	12	22	MAF	11	12	22	MAF	11	12	22	MAF	11	12	22	MAF	11	12	22	MAF	11	12	22	MAF	11	12	22	MAF
rs901104	G/A	424	150	14	0.151	399	174	14	0.172	390	140	19	0.162	966	366	34	0.159	418	151	13	0.152	266	92	2	0.133	435	143	9	0.137	148	58	3	0.153
rs1385600	A/G	419	154	16	0.158	388	183	16	0.183	388	143	18	0.163	954	369	35	0.162	409	153	22	0.169	256	101	4	0.151	429	147	12	0.145	131	67	10	0.209
rs1007837	T/C	422	151	16	0.155	396	177	14	0.175	390	134	18	0.157	968	363	32	0.157	411	146	23	0.166	259	97	5	0.148	433	140	14	0.143	127	72	9	0.216
rs4945261	G/A	424	150	13	0.150	397	174	16	0.175	390	138	18	0.159	960	370	32	0.159	415	153	13	0.154	264	94	2	0.136	433	143	10	0.139	148	56	4	0.154
rs7101429	A/G	425	150	13	0.150	397	174	16	0.175	391	139	18	0.160	960	371	32	0.160	415	151	13	0.153	265	93	2	0.135	434	142	10	0.138	148	56	4	0.154
rs4291702	C/T	418	154	15	0.157	394	177	16	0.178	389	137	18	0.159	958	359	36	0.159	407	155	20	0.168	256	97	4	0.147	430	144	13	0.145	132	66	10	0.207
rs7115850	G/C	414	159	16	0.162	390	182	15	0.181	385	144	16	0.161	931	379	37	0.168	394	164	20	0.176	255	97	6	0.152	423	150	14	0.152	129	69	10	0.214
rs2373115	C/A	409	161	15	0.163	385	185	16	0.185	384	145	16	0.162	932	380	35	0.167	397	165	19	0.175	254	97	6	0.153	421	149	15	0.152	130	68	7	0.200

Alleles;major/minor allele.

11; number of major allele homozygotes, 12; number of heterozygotes, 22; number of minor allele homozygotes.

MAF; minor allele frequency.

**Table 3:** Single variant association of eight *GAB2* variants with LOAD in the NCRAD series.

Variant	Min Allele	NCRAD *ε*4+/–			NCRAD *ε*4+			NCRAD *ε*4–		
		OR	95% CI	p	OR	95% CI	p	OR	95% CI	p
rs901104	A	0.86	0.59–1.26	0.44	1.06	0.64–1.76	0.81	0.73	0.45–1.18	0.20
rs1385600	G	0.79	0.58–1.08	0.14	0.60	0.33–1.12	0.11	0.84	0.60–1.16	0.28
rs1007837	C	0.76	0.56–1.05	0.09	0.60	0.33–1.10	0.10	0.81	0.57–1.13	0.21
rs4945261	A	0.96	0.74–1.25	0.78	0.97	0.59–1.58	0.90	0.95	0.69–1.30	0.74
rs7101429	G	0.96*	0.74–1.25	0.76	0.96*	0.59–1.58	0.89	0.95*	0.69–1.30	0.74
rs4291702	T	0.78	0.57–1.06	0.12	0.57	0.31–1.03	0.06	0.84	0.61–1.16	0.29
rs7115850	C	0.82	0.61–1.10	0.18	0.64	0.35–1.17	0.15	0.86	0.62–1.18	0.34
rs2373115	A	1.03	0.87–1.22	0.72	0.83	0.47–1.45	0.51	1.08	0.88–1.33	0.48

OR; odds ratio, 95%CI; 95% confidence intervals for binary logistic regression adjusted for *APOE ε*4 dose, sex and age-at-diagnosis.

* Schjeide *et al* published association of rs7101429, with LOAD in samples obtained from NCRAD [34]; although we have no way of ascertaining the level of sample overlap between these studies the different ORs reported in that publication (*ε*4+/–; 0.70, *ε*4+; 0.70, *ε*4–; 0.74) suggests our study contains some novel samples.

Since Reiman *et al* observed stronger associations in their *APOE ε*4+ subgroup, we also show the genotype counts (Table S1) and association results (Table 3) for the NCRAD *APOE ε*4+ and *ε*4– negative subgroups respectively. As shown in Table 3, unlike the associations reported by Reiman et al., there was no association with LOAD in the *ε*4+ or *ε*4– individuals (all p>0.06). We also tested for association of these eight variants in the *APOE ε*4 subgroups for our other case-controls series (JS, RS and AUT), however these data revealed no significant association (all p>0.05; data not shown).

### The *GAB2* locus shows significant heterogeneity across populations in a meta-analysis of 12,000+ samples

We next added our data to the available published data (ranging from **11,952 to 22,253** samples) to determine whether the significant association reported on Alzgene (www.alzgene.org) would survive the addition of 4,854 samples (22–40% increase in sample size), that showed no association individually). As shown in Figure 1, despite significant population heterogeneity across the series for all variants (all p<0.007), the GAB2 association with LOAD remained for all variants using the combined random effects model (all p<0.04).

### Association of *GAB2* variants with LOAD is dependent upon on haplotypic background

The comparable frequency (13.3%–20.9%) and effect size (OR = 0.79–0.88) that we report for the *GAB2* variants are likely due to the substantial linkage disequilibrium within the *GAB2* region (all pair-wise r^2^ >0.91). In order to characterize further the association of *GAB2* variants with LOAD, haplotypes were constructed for the eight variants as well as rs10793294 for which we had genotype data available (Table 4). These nine variants, spanning 161 kb of *GAB2* (202 kb), comprised three haplotypes with a frequency >1% in the RS, JS and AUT series and four haplotypes in the NCRAD series. This additional low frequency haplotype in the NCRAD series further highlights the heterogeneity that we observed for those samples in the single variant analyses.

**Table 4:** Association of *GAB2* haplotypes with LOAD.

Composition of the haplotypes										Haplotype frequencies (%LOAD:;%CTRL) by series (N LOAD: N CTRL)					Haplotype association (NCRAD)			
	rs901104	rs1385600	rs1007837	rs4945261	rs7101429	rs10793294	rs4291702	rs7115850	rs2373115	All (2030∶2407)	JS (578∶584)	RS (521∶1282)	AUT (559∶338)	NCRAD (372∶203)	OR	L95	U95	p-value
H1	0	0	0	0	0	0	0	0	0	77.3∶76.5	76.9∶74.6	76.9∶78.1	75.5∶78.4	80.3∶69.1	1.908	1.339	2.718	0.0003
H2	1	1	1	1	1	1	1	1	1	14.0∶14.5	14.1∶16.2	14.8∶14.5	14.3∶11.5	12.6∶14.3	0.805	0.517	1.255	0.34
H3	0	0	0	0	0	1	0	0	0	5.6∶5.4	5.7∶5.7	6.4∶4.9	6.1∶5.9	3.8∶6.5	0.519	0.254	1.061	0.07
H4	0	1	1	0	0	1	1	1	1	<1.0*	<1.0*	<1.0*	<1.0*	0.7∶3.2	0.214	0.061	0.750	0.02
H5	0	0	0	0	0	0	0	1	1	<1.0*	<1.0*	<1.0*	<1.0*	1.0∶1.0	2.100	0.549	8.035	0.28
Global p-value										0.18	0.42	0.47	0.13	0.0004				

The Haplotype columns show the allelic composition of each haplotype in the 5’ to 3’ orientation from the p to the q telomere of chromosome 11. 0; major allele, 1; minor allele.

Haplotypes are numbered according to their frequency. Only haplotypes with frequency >1% are shown.

OR; Odds ratio, L95; lower 95% confidence interval, U95; upper 95% confidence interval for association of the individual haplotypes in the NCRAD series.

* Due to the haplotype frequency cut-off (>1%) used in this study H4 was not included in the global analysis for the total, JS, RS or AUT series.

We performed global tests for haplotypic association with LOAD risk in each case-control series thereby reducing the number of tests performed. The haplotype frequencies and global haplotype p-values for each series are shown in Table 4. Consistent with the single variant results, the only series to show association was NCRAD (Global p-value = 0.0001). Individual haplotype score tests were subsequently calculated for NCRAD revealing that the most common haplotype (H1), which comprised the major allele at all nine variants, was present at an increased frequency in LOAD (79.8%, n = 1096) compared to control (69.0%, n = 280) chromosomes (OR = 1.86, p = 7.93×10^−5^). This is comparable to the findings reported by Reiman *et al* in their *APOE* ε4+ series (Discovery cohort; 76% LOAD, 68% controls, OR = 1.39, p = 0.05). Reflecting the strong linkage disequilibrium in this region, the second most common haplotype (H2) is comprised of the minor allele at all nine variants. Although a trend towards an opposing effect compared to H1 was observed (OR = 0.84), the association was not significant (p = 0.39). The third haplotype (H3) comprised the same alleles as H1 with the exception of rs10793294, for which we have previously published a significant protective association with LOAD [23]. Consistent with these previous findings, possession of rs10793294 on the H1 background resulted in a trend towards decreased risk (OR = 0.58, p = 0.07) compared to the risk association of H1 (OR = 1.86).

The haplotype frequencies observed here and the lack of association of H2 and H3 with LOAD are comparable to the findings of Reiman *et al*. In addition, we also observed a novel observation in the NCRAD series where a fourth haplotype (H4) was present at an increased frequency in controls (3.2%) compared to the other series such that it surpassed the 1% frequency cut-off for analysis. This haplotype, present in 8 (0.6%) LOAD compared to 13 (3.2%) control chromosomes, was associated with decreased risk for LOAD (OR = 0.17, p = 0.003), consistent with the fact that it comprises the five protective alleles observed in the single variant tests (Figure 1) as well as the protective rs10793294 allele [23]. Since Reiman *et al* used a haplotype frequency cut-off >5% they did not include this relatively rare haplotype in their analyses and so we are unable to ascertain whether the control samples in that study also had an increased frequency of this haplotype as observed in the NCRAD series. Since H4 was the only protective haplotype in these data (p<0.05), these findings indicate that a complex interaction of multiple functional variants across *GAB2* haplotypes is required to confer protection against LOAD rather than possession of any single *GAB2* variant. Notably, a search for conserved sequence revealed that rs901104 (71%), rs7115850 (95%) and rs2373115 (90%) lay in regions >70% conserved between human and mouse genomes making these three variants strong candidates for functional studies.

### Epistatic interaction exists between *GAB2* and *APOE* variants

In order to determine whether any genetic interaction exists between the *GAB2* and other strong LOAD candidates to modify LOAD risk, we tested for pair-wise epistatic interaction between the nine *GAB2* variants studied here and the strongest known LOAD risk factor, *APOE* ε4, as well as the top GWAS-identified variants for which we had genotype information available; *BIN1* (rs744373), *CLU* (rs11136000), *CR1* (rs3818361) and *PICALM* (rs3851179). The results for all 105 tests performed for each of the interactions are shown in Table S2. While we report 17 interactions (all *APOE*-*APOE, GAB2-GAB2* or *GAB2*-*APOE*) at the p≤0.05 level (Table 5), the only interaction that would survive Bonferroni correction for the 105 tests performed (p<0.0005) is the interaction between the two *APOE* variants that confer ApoE allelic status (p = 4.1×10^−12^). Four *GAB2*-*APOE* interactions surpassed the p<0.05 cut-off; *APOE* rs7412 × *AB2* rs10793294 (p = 0.016), rs1008737 (p = 0.026), rs7115850 (p = 0.045), rs4291702 (p = 0.049). Due to the multiple testing inherent in these analyses, further investigation of these possible epistatic interactions in multiple, independent studies is required in order to determine whether there is true synergy between the variants.

**Table 5:** Pair-wise epistatic interaction tests between variants in *GAB2* and *APOE*.

Variant 1			Variant 2			Interaction		
Chr	Gene	Variant	Chr	Gene	Variant	OR	Chi^2^	p-value
19	*APOE*	rs429358	19	*APOE*	rs7412	1.96	48.09	4.1×10^−12^
11	*GAB2*	rs1007837*	11	*GAB2*	rs2373115*	1.32	6.99	0.008
11	*GAB2*	rs1385600*	11	*GAB2*	rs1007837*	1.30	6.03	0.014
11	*GAB2*	rs10793294	19	*APOE*	rs7412	0.85	5.85	0.016
11	*GAB2*	rs1007837*	19	*APOE*	rs7412	0.85	4.93	0.026
11	*GAB2*	rs1007837*	11	*GAB2*	rs7101429	1.28	4.87	0.027
11	*GAB2*	rs1007837*	11	*GAB2*	rs7115850*	1.25	4.80	0.028
11	*GAB2*	rs1007837*	11	*GAB2*	rs4945261	1.27	4.76	0.029
11	*GAB2*	rs1385600*	11	*GAB2*	rs2373115*	1.25	4.62	0.032
11	*GAB2*	rs901104	11	*GAB2*	rs1007837*	1.26	4.29	0.038
11	*GAB2*	rs1385600*	11	*GAB2*	rs7101429	1.25	4.19	0.041
11	*GAB2*	rs1385600*	11	*GAB2*	rs7115850*	1.23	4.19	0.041
11	*GAB2*	rs1385600*	11	*GAB2*	rs4945261	1.25	4.07	0.044
11	*GAB2*	rs7115850*	19	*APOE*	rs7412	0.86	4.02	0.045
11	*GAB2*	rs4291702*	11	*GAB2*	rs2373115*	1.23	4.01	0.045
11	*GAB2*	rs1007837*	11	*GAB2*	rs4291702*	1.23	3.97	0.046
11	*GAB2*	rs4291702*	19	*APOE*	rs7412	0.86	3.88	0.049

Pair-wise interactions between fifteen variants in *GAB2*, *APOE*, *BIN1*, *CLU*, *CR1* and *PICALM* (105 tests) were performed. Interactions that gave a p-value <0.05 are shown in the table. The chromosome (Chr), gene and variant rs number for each interaction are given under the headings “Variant 1” and “Variant 2”. The odds ratio (OR), Chi^2^ value and p-value for the interaction test are shown for each pair-wise test. *associated with decreased risk in the NCRAD series.

### Protective GAB2 variants are associated with increased *GAB2* mRNA levels in lymphoblastoid cell lines

In an attempt to identify whether any of these *GAB2* variants could be associated with altered *GAB2* expression, we performed an *in silico* search of the data published by Dixon *et al* [24] in which they genotyped 498,273 variants across the human genome and measured quantitative expression of 54,675 transcripts representing 20,599 genes in Epstein-Barr virus-transformed lymphoblastoid cell lines (LCLs) derived from peripheral blood lymphocytes taken from 400 children. As shown in Table 6, although only three (rs1385600, rs4945261 and rs2373115) of the nine *GAB2* variants described here were included on the two genotyping platforms used by Dixon *et al*, all three were associated (p = 9.5×10^−7^, 9.3×10^−6^ and 9.3×10^−6^ respectively) with one of the six *GAB2* expression probes tested (1566958_at). Data pertaining to the association of these variants with the other five *GAB2* probes was not available indicating that the association fell below the cut-off LOD score (LOD>2) for inclusion in the database. The negative effect sizes shown in Table 6 indicate decreased *GAB2* expression associated with the major allele or inversely, increased *GAB2* expression associated with the minor allele in LCLs. Notably, the minor allele of all three of these variants were associated with decreased risk for LOAD in our NCRAD series. Therefore, variants associated with increased *GAB2* transcript (in LCLs) are also associated with decreased risk for LOAD.

**Table 6:** *GAB2* variants are associated with *GAB2* mRNA expression in lymphoblastoid cells.

ProbeID	Variant	Allele	Effect	LOD	p-value
1566958_at	rs1385600	Maj	−0.444	5.218	9.5×10^−7^
1566958_at	rs4945261	Maj	−0.451	4.266	9.3×10^−6^
1566958_at	rs2373115	Maj	−0.442	5.145	1.1×10^−6^

Data obtained from database published by Dixon *et al*.

ProbeID; *GAB2* cRNA probe ID (Affymetrix),

Allele; Major allele was tested in this analysis;

Effect; coefficient for linear regression model;

LOD; Logarithm of odds (threshold for genome-wide significance >6.076, equivalent to p<0.05).

In order to determine whether the variants were associated with altered *GAB2* expression in a tissue more directly relevant to LOAD, we measured *GAB2* mRNA levels in 356 cerebellum samples and 163 temporal cortex samples obtained from our autopsy-confirmed series (AUT) of LOAD patients and controls. ΔC_T_ GAB2 levels measured for all samples are available in Table S3. To decrease the number of statistical tests performed we tested for global association of the *GAB2* haplotypes with *GAB2* mRNA levels (one test) rather than testing each individual variant (9 tests). As shown in Table 7 we observed no global haplotypic association with *GAB2* mRNA levels in either temporal cortex or cerebellum regions in the LOAD patients or controls or when the two groups were pooled (all global p>0.43). Therefore, these data from the cerebellum and temporal cortex of LOAD patients (mean age = 74) and controls (mean age = 72) did not replicate the association of protective *GAB2* variants with increased *GAB2* transcript levels observed in the peripheral blood lymphocytes of children.

**Table 7:** *GAB2* haplotypes are not associated with *GAB2* mRNA expression in post-mortem brains.

Brain Region	Diagnosis	N	Global p-value	Haplotype (freq)
Temporal Cortex	LOAD	85	0.85	H1 (0.80), H2 (0.11), H3 (0.03), H5 (0.01), H4 (0.01)
Temporal Cortex	CTRL	78	0.85	H1 (0.80), H2 (0.11), H3 (0.03), H5 (0.01), H4 (0.01)
Temporal Cortex	ALL	163	0.67	H1 (0.80), H2 (0.11), H3 (0.04), H4 (0.01)
Cerebellum	LOAD	189	0.43	H1 (0.78), H2 (0.15), H3 (0.03), H4 (0.008)*
Cerebellum	CTRL	167	0.52	H1 (0.78), H2 (0.13), H3 (0.05), H5 (0.02), H4 (0.009)*
Cerebellum	ALL	356	0.46	H1 (0.78), H2 (0.14), H3 (0.04), H5 (0.01), H4 (0.008)*

N; number of individuals included in the analysis.

Global p-value; global association of *GAB2* haplotypes with *GAB2* mRNA expression in post-mortem temporal cortex and cerebellum samples.

A novel haplotype (H5) comprising the major allele at all variants except rs7115850 and rs2373115 exceeded the cut-off frequency and was observed at a higher frequency than H4 in four of these sample subgroups.

* Due to the haplotype frequency cut-off (>1%) used in this study H4 was not included in the global analysis for these subgroups.

### Increased *GAB2* mRNA levels in the postmortem temporal cortex are associated with decreased AD pathology

We next assessed whether *GAB2* was differentially expressed in the postmortem temporal cortex and cerebellum samples from LOAD and control patients. The number of pathologial markers (cortical neurofibrillary tangles and senile plaques) counted for each sample is shown in Table S3. As shown in Table 8, while *GAB2* mRNA levels did not significantly differ between LOAD and control brains in the cerebellum (two-tailed p = 0.26), increased *GAB2* mRNA levels were measured in the temporal cortex of control versus LOAD brains (two-tailed p = 0.0006) suggesting the possibility that increased *GAB2* expression could be protective against (or down-regulated in response to) LOAD. Alternatively, it is possible that *GAB2* mRNA expression is acting as a proxy for another confounding variable. To investigate this, we tested for differences in RNA integrity (RNA integrity number or RIN), age-at-death, *APOE ε*4 dose and sex between LOAD and control samples (Table 8). We found no difference in RIN (cerebellum one-sided p = 0.59; temporal cortex one-sided p = 0.89) thereby demonstrating that the integrity of the RNA was not affected by the presence of AD pathology. We did however find that the LOAD brains on average were taken from older individuals (mean age difference  = 2.2 years; p = 0.002) from a greater number of *APOE ε*4 carriers (36% more versus controls; p<0.0001) and a greater number of females (15% more versus controls; p = 0.02).

**Table 8:** *GAB2* mRNA expression is increased in temporal cortex of control compared to LOAD brains.

Variable	LOAD (n = 128)	CTRLs (n = 121)	LOAD + CTRLs (n = 249)	p-value
*GAB2* mRNA Cerebellum	N = 127;Mean = 1.7+/−0.1	N = 118;Mean = 1.8;+/−0.1	N = 245;Mean = 1.7+/−0.1	0.26
*GAB2* mRNA Temporal cortex	N = 59;Mean = 0.8+/−0.1	N = 43;Mean = 1.3;+/−0.1	N = 102;Mean = 1.0+/−0.1	0.0006
RIN Cerebellum	N = 127;Mean = 7.2+/−0.1	N = 118;Mean = 7.1+/−0.1	N = 245;Mean = 7.2+/−0.1	0.59
RIN Temporal Cortex	N = 59;Mean = 6.8+/−0.1	N = 43;Mean = 6.8+/−0.1	N = 102;Mean = 6.8+/−0.1	0.89
Age-at-death (yrs)	N = 128;Mean = 73.9+/−0.5	N = 121;Mean = 71.7+/−0.5	N = 249;Mean = 72.8+/−0.3	0.002
*APOE* 4 (n for 0,1,2 copies)	N = 126;(52,58,16)	N = 119;(92,26,1)	N = 249;(148,84,17)	<0.0001
Sex (M,F)	N = 128;(62,66)	N = 121;(77,44)	N = 249;(139,110)	0.02
Neurofibrillary tangles	N = 128;Mean = 11.7+/−0.5	N = 121;Mean = 0.1+/−0.1	N = 249;Mean = 6.1+/−0.5	<0.0001
Senile plaques	N = 128;Mean = 42.9+/−0.5	N = 121;Mean = 6.3+/−0.9	N = 249;Mean = 25.1+/−1.3	<0.0001

N; number of brains analysed,

Mean; mean value +/− standard deviation.

p-value; for independent t-test or chi-squared test (*APOE ε*4 dose and sex) for variable in LOAD versus control brains.

We next tested for a correlation between *GAB2* mRNA levels and RIN, age-at-death, *APOE ε*4 dose, sex and postmortem pathology. To increase the statistical power we tested for association in all post-mortem brains (LOAD and controls). As shown in Table 9, we found that RIN (r = 0.68, p<0.0001), sex (r = −0.28, p = 0.005), number of NFTs (r = −0.34, p = 0.0006) and number of senile plaques (r = −0.32, p = 0.001) were the best predictors of *GAB2* mRNA levels in the temporal cortex whereas RIN (r = 0.76, p<0.0001) was the best predictor in cerebellum (a brain region much less affected by AD pathology than the cortex). Although the NFT and senile plaque counts were strongly correlated with each other (r = 0.84, p<0.0001), neither were correlated with RIN for RNA extracted from the temporal cortex (r = −0.06, p = 0.29 and r = −0.05, p = 0.31, respectively) indicating that the correlation of plaque and tangle count with cortical *GAB2* levels cannot be attributed to by increased RNA integrity of samples with less pathology. On the other hand the NFT (r = 0.20, p = 0.0009) and senile plaque counts (r = 0.19, p = 0.001) were higher in female brains thus indicating that sex maybe a contributing factor to the association of increased NFT and senile plaque counts with decreased cortical *GAB2* levels.

**Table 9:** Increased *GAB2* mRNA expression is associated with decreased AD pathology in temporal cortex.

	Temporal cortex (n = 102)				Cerebellum (n = 245)			
Variable	Co-efficient	95% CI		p-value	Co-efficient	95% CI		p-value
RNA integrity number	0.675	0.55	0.77	<0.0001	0.756	0.69	0.81	<0.0001
Age-at-death (yrs)	−0.078	−0.27	0.12	0.43	−0.093	−0.22	0.04	0.15
*APOE* 4 dose (0<1<2 copies)	−0.084	−0.28	0.12	0.40	−0.077	−0.20	0.05	0.23
Sex (M<F)	−0.275	−0.45	−0.08	0.005	−0.115	−0.24	0.01	0.07
Number of Neurofibrillary tangles	−0.336	−0.50	−0.15	0.0006	−0.071	−0.19	0.06	0.27
Number of Senile plaques	−0.321	−0.49	−0.13	0.001	−0.097	−0.22	0.03	0.13

Co-efficient; Spearman's rank correlation coefficient, 95%CI; confidence intervals for correlation coefficient, p-value; significance level.

## Discussion

Successful replication of candidate genes for complex diseases in multiple, large, independent case-control series are invaluable for determining true risk loci from false-positive associations. Once genetic involvement in the disease has been well established, functional studies can then be used to assess the biochemical properties of the protein with the aim of identifying putative therapeutic targets. Here, we have performed a large follow-up case-control association study for *GAB2* and revealed significant association in one out of the four populations studied for five *GAB2* variants (0.0008<p<0.04). However, this positive association must be treated with caution due to the heterogeneity observed compared to the other three homogenous populations studied. The reason for the disparate association in the NCRAD series could be due to the fact that it is the population with the fewest controls and therefore more susceptible to inflated effect sizes, population substructure or genotyping error. Nevertheless, similar frequencies were also reported to Reiman et al., highlighting the possibility that there is true population heterogeneity at this locus. Based on the fact that meta-analysis of the four populations did not reveal association of any of the variants we can only conclude that our data do not support the genetic association of *GAB2* with LOAD. As a testament to the increased statistical power achieved by analyzing multiple, independent case-control series, meta-analyses for the *GAB2* variants combining our data with all available previously published data (ranging from 11,952 to 22,253 samples) revealed significant association for all nine variants (all p<0.04) despite significant population heterogeneity and the fact that 22−40% of the samples did not show association when tested independently.

Investigation of the haplotypic association of *GAB2* with LOAD risk revealed that the relatively rare H4 haplotype (which comprises the five variants that conferred protection against LOAD in our NCRAD series and a protective variant we have reported previously) was observed at an increased frequency in NCRAD controls (3.2%) compared to NCRAD LOAD patients (0.6%) and also compared to other control populations (<1%) indicating that inheritance of these six protective alleles together is usually rare but when it does occur, it may protect against LOAD. The fact that H2, a more frequent haplotype (13% LOAD, 14.3% controls) also comprises the minor allele at these six variants (in addition to the minor alleles at the other three) but only trends towards a protective association in the same population (p = 0.39) suggests that the protection associated with H4 in the NCRAD series is not merely due to possession of the minor allele at these six variants (present in H2 and H4) but also to the major allele at the other three (H4). In contrast, lacking the protective alleles (as is the case in the most common haplotpye, H1), appears to be sufficient to confer risk for LOAD despite possession of the major alleles at the other three variants (H1 and H4). These findings provide support for the hypothesis that susceptibility to LOAD is dependent on the *GAB2* haplotypic background rather than to possession of a single functional allele. We have identified two of these protective variants (rs7115850, rs2373115) worthy of prioritized follow-up functional investigation based on their location within conserved regions between human and mouse genomes. It must be noted that according to Tagger (a bioinformatic tool for the selection and evaluation of tag SNPs from genotype data [29]), the nine variants analysed here belong to three of the seven linkage blocks that comprise *GAB2*. It is therefore possible that other haplotypes at the *GAB2* locus that were not covered by the variants in this study also contribute to LOAD risk.

Here we have also shown that all three *GAB2* variants included in a dataset published by Dixon *et al* were associated with increased *GAB2* mRNA levels in LCLs derived from lymphocytes taken from children (all p<9.3×10^−6^); two of these variants were protective in our NCRAD series. In summary, variants that conferred protection for LOAD in our NCRAD series and in meta-analysis of all published data were associated with increased *GAB2* mRNA levels in LCLs, indicating that increased *GAB2* levels may be protective against LOAD.

We next tested for association of *GAB2* variants with *GAB2* mRNA levels brain tissue obtained at autopsy to improve detection of LOAD-related *GAB2* variant/transcript associations that might go undetected in the peripheral tissue lymphoblastoid cell lines. We analysed brain tissue from the temporal cortex, the most affected brain region in LOAD but because LOAD causes profound neuronal cell loss and astrogliosis that will alter mRNA levels in affected regions, mRNAs were also measured in the cerebellum which is largely unaffected by LOAD pathology. Although the single variants trended towards the same association observed in the LCLs we did not observe any significant association in postmortem cerebellum or temporal cortex samples from control and LOAD brains. One possible explanation for this is that mRNAs are degraded in the postmortem interval and are likely to be influenced by the agonal state prior to death, which could alter the *GAB2* transcript level. The Dixon *et al* study measured *GAB2* transcript levels in lymphocytes taken from living children and so would have been unaffected by the agonal state. Overall, there are many possible explanations for the lack of replication observed between LCLs and postmortem samples that include but are not limited to the lack of power to detect the association in postmortem tissue, tissue-specific, age-related or disease-specific expression levels of *GAB2* mRNA.

The assertion that increased *GAB2* expression levels may protect against LOAD is not novel. Other studies have shown that Gab2 protein is detected in AD brains with the highest levels found in some of the most affected AD areas such as the hippocampus and cingulate gyrus within highly dystrophic neurons containing neurofibrillary tangles, which along with senile plaques, are a pathological hallmark of AD [12]. Furthermore, Reiman *et al* showed that *GAB2* siRNA treatment was associated with a 1.70-fold increase in hyper-phosphorylated tau, the principal component of neurofibrillary tangles [12]. Based on this finding and along with the fact that Gab2 is the principal activator of the phosphatidylinositol 3-kinase signaling pathway [30], activation of which suppresses glycogen synthase kinase 3-mediated phosphorylation of tau and prevents apoptosis of confluent cells [31], Reiman *et al* hypothesized that Gab2 might function to protect neurons from neurofibrillary tangle formation and that a loss-of-function *GAB2* haplotype would increase tau phosphorylation at sites abnormally phosphorylated in AD brains. Consistent with this hypothesis, we have observed a correlation between increased *GAB2* mRNA levels in postmortem temporal cortex and decreased neurofibrillary tangle counts (p = 0.0006) and decreased senile plaque counts (p = 0.001). No association was observed in the cerebellum. Since the temporal cortex is more affected by AD pathology than the cerebellum it is reasonable to assume that due to regional specific cell death, the underlying distribution of cells would be different between the two areas, which could explain why we see less *GAB2* expression in the cortex (mean ΔC_T_  = 1.0) versus the cerebellum (mean ΔC_T_  = 1.7) and why we see an association of *GAB2* expression with AD pathology in the predominantly pathology-affected area only. Taken together with our observation that *GAB2* variants associated with decreased risk for LOAD may increase *GAB2* mRNA levels (data taken from LCLs not the cortex), the correlation of increased *GAB2* mRNA with decreased NFT and senile plaque counts in a tissue directly affected by LOAD provides further support for the hypothesis that Gab2 may protect neurons from LOAD pathology.

In summary, we have used a joint analysis approach to identify biologically congruent associations between genetic association and gene expression levels. We have identified a strong association of *GAB2* gene expression levels with neurofibrillary tangle and senile plaque counts in temporal cortex using only 102 subjects and shown that despite the fact that three of our four case-control series did not replicate the previously reported evidence that *GAB2* variants protect against LOAD, our meta-analyses of 11,952-22,253 samples from this study and those published still showed a strong association at this locus. Finally we have provided evidence that these protective variants may functionally increase *GAB2* gene expression. We recently used a similar approach to identify functional variants in the insulin degrading enzyme that conferred protection against LOAD [32], [33] thus providing further support for multi-platform approaches to investigate candidate genes for complex diseases such as LOAD.

## Supporting Information

Text S1 **Genetic and Environmental Risk for Alzheimer’s disease (GERAD1) Consortium Author List and Affiliations.**
(DOC)

Table S1 **Genotype, allele counts and allele frequencies for the eight** ***GAB2*** **variants.**
(DOC)

Table S2 **Pair-wise epistatic interaction tests between variants in** ***GAB2*** **,** ***APOE*** **,** ***BIN1*** **,** ***CLU*** **,** ***CR1*** **and** ***PICALM*** **.**
(DOC)

Table S3 **GAB2 mRNA levels and postmortem pathology.**
(DOC)
