# Supplementary material for: Linking Protective GAB2 Variants, Increased Cortical GAB2 Expression and Decreased Alzheimer’s Disease Pathology
Source: PLoS One. 2013 May 28;8(5):e64802. doi: 10.1371/journal.pone.0064802 (PMC3665686; doi:10.1371/journal.pone.0064802)
Supplement: Text S1 — Genetic and Environmental Risk for Alzheimer’s disease (GERAD1) Consortium Author List and Affiliations. (DOC) [file pone.0064802.s001.doc]

**Text S1**

**Genetic and Environmental Risk for Alzheimer’s disease (GERAD1) Consortium Author List**

Denise Harold^1^, Rebecca Sims^1^, Amy Gerrish^1^, Jade Chapman^1^, Valentina Moskvina^1^, Richard Abraham^1^, Paul Hollingworth^1^, Marian Hamshere^1^, Jaspreet Singh Pahwa^1^, Kimberley Dowzell^1^, Amy Williams^1^, Nicola Jones^1^, Charlene Thomas^1^, Alexandra Stretton^1^, Angharad Morgan^1^, Kate Williams^1^, Simon Lovestone^2^, John Powell^2^, Petroula Proitsi^2^, Michelle K Lupton^2^, Carol Brayne^3^, David C. Rubinsztein^4^, Michael Gill^5^, Brian Lawlor^5^, Aoibhinn Lynch^5^, Kevin Morgan^6^, Kristelle Brown^6^, Peter Passmore^7^, David Craig^7^, Bernadette McGuinness^7^, Janet A Johnston^7^, Stephen Todd^7^, Clive Holmes^8^, David Mann^9^, A. David Smith^10^, Seth Love^11^, Patrick G. Kehoe^11^, John Hardy^12^, Rita Guerreiro^13,33^, Andrew Singleton^13^, Simon Mead^14^, Nick Fox^15^, Martin Rossor^15^, John Collinge^14^, Wolfgang Maier^16^, Frank Jessen^16^, Reiner Heun^16^, Britta Schürmann^16,17^, Alfredo Ramirez^16^, Christine Herold^34^, André Lacour^34^, Dmitriy Drichel^34^, Hendrik van den Bussche^18^, Isabella Heuser^19^, Johannes Kornhuber^20^, Jens Wiltfang^21^, Martin Dichgans^22,23^, Lutz Frölich^24^, Harald Hampel^25^, Michael Hüll^26^, Dan Rujescu^27^, Alison Goate^28^, John S.K. Kauwe^29^, Carlos Cruchaga^28^, Petra Nowotny^28^, John C. Morris^28^, Kevin Mayo^28^, Gill Livingston^30^, Nicholas J. Bass^30^, Hugh Gurling^30^, Andrew McQuillin^30^, Rhian Gwilliam^31^, Panagiotis Deloukas^31^, Markus M. Nöthen^32^, Peter Holmans^1^, Michael O’Donovan^1^, Michael J.Owen^1^, Julie Williams^1^.

### Affiliations

^1^ Medical Research Council (MRC) Centre for Neuropsychiatric Genetics and Genomics, Neurosciences and Mental Health Research Institute, Department of Psychological Medicine and Neurology, School of Medicine, Cardiff University, Cardiff, UK.

^2^ King's College London, Institute of Psychiatry, Department of Neuroscience, De Crespigny Park, Denmark Hill, London.

^3^ Institute of Public Health, University of Cambridge, Cambridge, UK.

^4^ Cambridge Institute for Medical Research, University of Cambridge, Cambridge, UK.

^5^ Mercer's Institute for Research on Aging, St. James Hospital and Trinity College, Dublin, Ireland.

^6^ Institute of Genetics, Queen's Medical Centre, University of Nottingham, UK.

^7^ Ageing Group, Centre for Public Health, School of Medicine, Dentistry and Biomedical Sciences, Queen's University Belfast, UK.

^8^ Division of Clinical Neurosciences, School of Medicine, University of Southampton, Southampton, UK.

^9^ Clinical Neuroscience Research Group, Greater Manchester Neurosciences Centre, University of Manchester, Salford, UK.

^10^ Oxford Project to Investigate Memory and Ageing (OPTIMA), University of Oxford, Level 4, John Radcliffe Hospital, Oxford, UK.

^11^ University of Bristol Institute of Clinical Neurosciences, School of Clinical Sciences, Frenchay Hospital, Bristol, UK

^12^ Department of Molecular Neuroscience and Reta Lilla Weston Laboratories, Institute of Neurology, UCL, London, UK.

^13^ Laboratory of Neurogenetics, National Institute on Aging, National Institutes of Health, Bethesda, Maryland, United States of America

^14^ MRC Prion Unit, Department of Neurodegenerative Disease, UCL Institute of Neurology, London, UK.

^15^ Dementia Research Centre, Department of Neurodegenerative Diseases, University College London, Institute of Neurology, London, UK.

^16^ Department of Psychiatry, University of Bonn, Sigmund-Freud-Straβe 25, 53105 Bonn, Germany.

^17^ Institute for Molecular Psychiatry, University of Bonn, Bonn, Germany

^18^ Institute of Primary Medical Care, University Medical Center Hamburg-Eppendorf, Germany.

^19^ Department of Psychiatry, Charité Berlin, Germany.

^20^ Department of Psychiatry, University of Erlangen, Nürnberg, Germany.

^21^ LVR-Hospital Essen, Department of Psychiatry and Psychotherapy, University Duisburg-Essen, Germany.

^22^ Institute for Stroke and Dementia Reserach, Klinikum der Universität München, Marchioninistr. 15, 81377, Munich, Germany.

^23^ Department of Neurology, Klinikum der Universität München, Marchioninistr. 15, 81377, Munich, Germany.

^24^ Central Institute of Mental Health, Medical Faculty Mannheim, University of Heidelberg, Germany.

^25^ Department of Psychiatry, Psychosomatic Medicine and Psychotherapy, Goethe University, Frankfurt, Germany

^26^ Centre for Geriatric Medicine and Section of Gerontopsychiatry and Neuropsychology, Medical School, University of Freiburg, Germany.

^27^ Alzheimer Memorial Center and Geriatric Psychiatry Branch, Department of Psychiatry, Ludwig-Maximilian University, Munich, Germany

^28^ Departments of Psychiatry, Neurology and Genetics, Washington University School of Medicine, St Louis, MO 63110, US.

^29^ Department of Biology, Brigham Young University, Provo, UT, 84602, USA.

^30^ Department of Mental Health Sciences, University College London, UK.

^31^ The Wellcome Trust Sanger Institute, Wellcome Trust Genome Campus, Hinxton, Cambridge, UK.

^32^ Department of Genomics, Life & Brain Center, University of Bonn, Bonn, Germany

^33^ Department of Molecular Neuroscience, Institute of Neurology, University College London, Queen Square, London WC1N 3BG, UK

^34^ Deutsches Zentrum für Neurodegenerative Erkrankungen (DZNE), Bonn
