# Supplementary material for: Linking Protective GAB2 Variants, Increased Cortical GAB2 Expression and Decreased Alzheimer’s Disease Pathology
Source: PLoS One. 2013 May 28;8(5):e64802. doi: 10.1371/journal.pone.0064802 (PMC3665686; doi:10.1371/journal.pone.0064802)
Supplement: Table S1 — Genotype, allele counts and allele frequencies for the eight GAB2 variants. (DOC) [file pone.0064802.s002.doc]

**Table S1. Genotype, allele counts and allele frequencies for the eight *GAB2* variants.** A) *APOE ε*4+ and B) *APOE ε*4- individuals. Maj=major allele; Min=minor allele; 11=major allele homozygote, 12=heterozygote, 22=minor allele homozygote; MAF=minor allele frequency.

**A)**

**B)**
