# Supplementary material for: Linking Protective GAB2 Variants, Increased Cortical GAB2 Expression and Decreased Alzheimer’s Disease Pathology
Source: PLoS One. 2013 May 28;8(5):e64802. doi: 10.1371/journal.pone.0064802 (PMC3665686; doi:10.1371/journal.pone.0064802)
Supplement: Table S2 — Pair-wise epistatic interaction tests between variants in GAB2 , APOE , BIN1 , CLU , CR1 and PICALM . (DOC) [file pone.0064802.s003.doc]

**Table S2. Pair-wise epistatic interaction tests between variants in *GAB2*, *APOE*, *BIN1*, *CLU*, *CR1*and *PICALM*.** Pair-wise interactions between the fifteen variants are shown. The chromosome (Chr), gene and variant rs number for each pair of variants are given under the headings “Variant 1” and “Variant 2”. The odds ratio (OR), Chi^2^ value and p-value for the interaction test are shown for each pair-wise test.

| **Variant 1** | | | **Variant 2** | | |  |  |  |
| --- | --- | --- | --- | --- | --- | --- | --- | --- |
| **Chr** | **Gene** | **Variant** | **Chr** | **Gene** | **Variant** | **OR** | **Chi^2^** | **p-value** |
| 19 | *APOE* | rs429358 | 19 | *APOE* | rs7412 | 1.96 | 48.09 | 4.07E-12 |
| 11 | *GAB2* | rs1007837 | 11 | *GAB2* | rs2373115 | 1.317 | 6.992 | 0.008187 |
| 11 | *GAB2* | rs1385600 | 11 | *GAB2* | rs1007837 | 1.295 | 6.032 | 0.01405 |
| 11 | *GAB2* | rs10793294 | 19 | *APOE* | rs7412 | 0.8483 | 5.852 | 0.01556 |
| 11 | *GAB2* | rs1007837 | 19 | *APOE* | rs7412 | 0.8465 | 4.929 | 0.02642 |
| 11 | *GAB2* | rs1007837 | 11 | *GAB2* | rs7101429 | 1.275 | 4.871 | 0.02732 |
| 11 | *GAB2* | rs1007837 | 11 | *GAB2* | rs7115850 | 1.254 | 4.803 | 0.02842 |
| 11 | *GAB2* | rs1007837 | 11 | *GAB2* | rs4945261 | 1.271 | 4.759 | 0.02914 |
| 11 | *GAB2* | rs1385600 | 11 | *GAB2* | rs2373115 | 1.247 | 4.618 | 0.03163 |
| 11 | *GAB2* | rs901104 | 11 | *GAB2* | rs1007837 | 1.26 | 4.288 | 0.03839 |
| 11 | *GAB2* | rs1385600 | 11 | *GAB2* | rs7101429 | 1.254 | 4.192 | 0.04062 |
| 11 | *GAB2* | rs1385600 | 11 | *GAB2* | rs7115850 | 1.231 | 4.191 | 0.04064 |
| 11 | *GAB2* | rs1385600 | 11 | *GAB2* | rs4945261 | 1.25 | 4.071 | 0.04363 |
| 11 | *GAB2* | rs7115850 | 19 | *APOE* | rs7412 | 0.8615 | 4.018 | 0.04502 |
| 11 | *GAB2* | rs4291702 | 11 | *GAB2* | rs2373115 | 1.229 | 4.011 | 0.04521 |
| 11 | *GAB2* | rs1007837 | 11 | *GAB2* | rs4291702 | 1.234 | 3.969 | 0.04634 |
| 11 | *GAB2* | rs4291702 | 19 | *APOE* | rs7412 | 0.8626 | 3.878 | 0.04893 |
| 11 | *GAB2* | rs1385600 | 19 | *APOE* | rs7412 | 0.8685 | 3.528 | 0.06034 |
| 11 | *GAB2* | rs901104 | 11 | *GAB2* | rs1385600 | 1.234 | 3.509 | 0.06104 |
| 8 | *CLU* | rs11136000 | 11 | *PICALM* | rs3851179 | 1.176 | 3.466 | 0.06263 |
| 11 | *GAB2* | rs1385600 | 11 | *GAB2* | rs4291702 | 1.211 | 3.371 | 0.06636 |
| 11 | *GAB2* | rs901104 | 11 | *GAB2* | rs7115850 | 1.217 | 3.219 | 0.07278 |
| 11 | *GAB2* | rs2373115 | 11 | *GAB2* | rs10793294 | 1.184 | 3.149 | 0.07598 |
| 11 | *GAB2* | rs1385600 | 11 | *GAB2* | rs10793294 | 1.187 | 2.962 | 0.08524 |
| 11 | *GAB2* | rs4945261 | 11 | *GAB2* | rs2373115 | 1.198 | 2.767 | 0.09625 |
| 11 | *GAB2* | rs901104 | 11 | *GAB2* | rs2373115 | 1.199 | 2.764 | 0.09642 |
| 11 | *GAB2* | rs4945261 | 11 | *GAB2* | rs7115850 | 1.196 | 2.736 | 0.0981 |
| 11 | *GAB2* | rs2373115 | 19 | *APOE* | rs7412 | 0.8847 | 2.651 | 0.1035 |
| 8 | *CLU* | rs11136000 | 11 | *GAB2* | rs10793294 | 0.8501 | 2.633 | 0.1046 |
| 2 | *BIN1* | rs744373 | 11 | *GAB2* | rs10793294 | 0.8435 | 2.552 | 0.1101 |
| 11 | *GAB2* | rs7101429 | 11 | *GAB2* | rs7115850 | 1.189 | 2.55 | 0.1103 |
| 11 | *GAB2* | rs7101429 | 11 | *GAB2* | rs2373115 | 1.183 | 2.411 | 0.1205 |
| 11 | *GAB2* | rs4291702 | 11 | *GAB2* | rs7115850 | 1.17 | 2.374 | 0.1234 |
| 11 | *GAB2* | rs901104 | 11 | *GAB2* | rs4291702 | 1.183 | 2.27 | 0.1319 |
| 11 | *GAB2* | rs1007837 | 11 | *GAB2* | rs10793294 | 1.166 | 2.248 | 0.1338 |
| 11 | *GAB2* | rs10793294 | 19 | *APOE* | rs429358 | 0.8671 | 2.21 | 0.1371 |
| 2 | *BIN1* | rs744373 | 11 | *GAB2* | rs2373115 | 0.8397 | 2.205 | 0.1376 |
| 11 | *GAB2* | rs7101429 | 11 | *GAB2* | rs4291702 | 1.179 | 2.177 | 0.1401 |
| 11 | *GAB2* | rs4945261 | 11 | *GAB2* | rs4291702 | 1.179 | 2.161 | 0.1416 |
| 1 | *CR1* | rs3818361 | 19 | *APOE* | rs429358 | 1.254 | 2.125 | 0.1449 |
| 11 | *GAB2* | rs901104 | 11 | *GAB2* | rs7101429 | 1.178 | 2.07 | 0.1502 |
| 11 | *GAB2* | rs901104 | 11 | *GAB2* | rs4945261 | 1.177 | 2.036 | 0.1536 |
| 11 | *GAB2* | rs4945261 | 11 | *GAB2* | rs10793294 | 1.161 | 1.893 | 0.1689 |
| 11 | *GAB2* | rs7101429 | 11 | *GAB2* | rs10793294 | 1.159 | 1.843 | 0.1746 |
| 11 | *GAB2* | rs7115850 | 11 | *GAB2* | rs2373115 | 1.149 | 1.795 | 0.1803 |
| 11 | *GAB2* | rs7115850 | 11 | *GAB2* | rs10793294 | 1.13 | 1.646 | 0.1995 |
| 11 | *GAB2* | rs901104 | 11 | *GAB2* | rs10793294 | 1.142 | 1.497 | 0.2211 |
| 1 | *CR1* | rs3818361 | 2 | *BIN1* | rs744373 | 0.8735 | 1.448 | 0.2288 |
| 11 | *PICALM* | rs3851179 | 19 | *APOE* | rs7412 | 1.088 | 1.411 | 0.2349 |
| 11 | *GAB2* | rs4291702 | 11 | *GAB2* | rs10793294 | 1.127 | 1.356 | 0.2442 |
| 2 | *BIN1* | rs744373 | 11 | *GAB2* | rs7115850 | 0.8719 | 1.352 | 0.2449 |
| 11 | *GAB2* | rs4945261 | 19 | *APOE* | rs429358 | 0.8806 | 1.298 | 0.2546 |
| 11 | *GAB2* | rs7101429 | 19 | *APOE* | rs429358 | 0.8808 | 1.289 | 0.2562 |
| 11 | *GAB2* | rs10793294 | 11 | *PICALM* | rs3851179 | 1.111 | 1.136 | 0.2865 |
| 11 | *GAB2* | rs4291702 | 19 | *APOE* | rs429358 | 0.8923 | 1.101 | 0.2941 |
| 8 | *CLU* | rs11136000 | 11 | *GAB2* | rs7115850 | 0.897 | 0.9841 | 0.3212 |
| 11 | *GAB2* | rs901104 | 19 | *APOE* | rs7412 | 0.9279 | 0.8728 | 0.3502 |
| 2 | *BIN1* | rs744373 | 11 | *GAB2* | rs1007837 | 0.8941 | 0.8643 | 0.3525 |
| 11 | *PICALM* | rs3851179 | 19 | *APOE* | rs429358 | 1.12 | 0.7995 | 0.3712 |
| 8 | *CLU* | rs11136000 | 11 | *GAB2* | rs2373115 | 0.908 | 0.761 | 0.383 |
| 11 | *GAB2* | rs7115850 | 19 | *GAB2* | rs429358 | 0.9117 | 0.7287 | 0.3933 |
| 11 | *GAB2* | rs901104 | 11 | *PICALM* | rs3851179 | 0.9087 | 0.6921 | 0.4054 |
| 2 | *BIN1* | rs744373 | 19 | *APOE* | rs7412 | 1.064 | 0.641 | 0.4234 |
| 11 | *GAB2* | rs4945261 | 11 | *PICALM* | rs3851179 | 0.9123 | 0.6406 | 0.4235 |
| 8 | *CLU* | rs11136000 | 11 | *GAB2* | rs1385600 | 0.9168 | 0.6265 | 0.4286 |
| 2 | *BIN1* | rs744373 | 11 | *GAB2* | rs1385600 | 0.909 | 0.6236 | 0.4297 |
| 1 | *CR1* | rs3818361 | 11 | *GAB2* | rs2373115 | 1.109 | 0.6021 | 0.4378 |
| 11 | *GAB2* | rs7101429 | 11 | *PICALM* | rs3851179 | 0.9154 | 0.5957 | 0.4402 |
| 2 | *BIN1* | rs744373 | 11 | *GAB2* | rs4291702 | 0.9156 | 0.5367 | 0.4638 |
| 8 | *CLU* | rs11136000 | 19 | *APOE* | rs7412 | 0.951 | 0.5249 | 0.4688 |
| 2 | *BIN1* | rs744373 | 11 | *GAB2* | rs7101429 | 0.9133 | 0.5237 | 0.4693 |
| 11 | *GAB2* | rs901104 | 19 | *APOE* | rs429358 | 0.9226 | 0.5115 | 0.4745 |
| 8 | *CLU* | rs11136000 | 11 | *GAB2* | rs1007837 | 0.9244 | 0.4957 | 0.4814 |
| 2 | *BIN1* | rs744373 | 11 | *GAB2* | rs901104 | 0.9175 | 0.4599 | 0.4977 |
| 1 | *CR1* | rs3818361 | 11 | *GAB2* | rs7101429 | 1.099 | 0.4556 | 0.4997 |
| 8 | *CLU* | rs11136000 | 11 | *GAB2* | rs4291702 | 0.9282 | 0.4501 | 0.5023 |
| 11 | *GAB2* | rs1385600 | 19 | *APOE* | rs429358 | 0.9301 | 0.4491 | 0.5028 |
| 11 | *GAB2* | rs7101429 | 19 | *APOE* | rs7412 | 0.9494 | 0.4262 | 0.5139 |
| 1 | *CR1* | rs3818361 | 11 | *GAB2* | rs4945261 | 1.094 | 0.4188 | 0.5175 |
| 8 | *CLU* | rs11136000 | 19 | *APOE* | rs429358 | 0.9227 | 0.4083 | 0.5229 |
| 2 | *BIN1* | rs744373 | 11 | *GAB2* | rs4945261 | 0.9274 | 0.3607 | 0.5481 |
| 11 | *GAB2* | rs4945261 | 19 | *APOE* | rs7412 | 0.9541 | 0.3487 | 0.5549 |
| 11 | *GAB2* | rs2373115 | 19 | *APOE* | rs429358 | 0.9377 | 0.3453 | 0.5568 |
| 1 | *CR1* | rs3818361 | 19 | *APOE* | rs7412 | 1.045 | 0.2866 | 0.5924 |
| 1 | *CR1* | rs3818361 | 11 | *GAB2* | rs4291702 | 1.068 | 0.2431 | 0.622 |
| 1 | *CR1* | rs3818361 | 11 | *GAB2* | rs901104 | 1.063 | 0.1946 | 0.6591 |
| 11 | *GAB2* | rs1007837 | 19 | *APOE* | rs429358 | 0.9583 | 0.1494 | 0.6991 |
| 1 | *CR1* | rs3818361 | 11 | *GAB2* | rs7115850 | 1.041 | 0.09139 | 0.7624 |
| 2 | *BIN1* | rs744373 | 8 | *CLU* | rs11136000 | 1.028 | 0.08364 | 0.7724 |
| 8 | *CLU* | rs11136000 | 11 | *GAB2* | rs901104 | 0.9676 | 0.07997 | 0.7773 |
| 11 | *GAB2* | rs7115850 | 11 | *PICALM* | rs3851179 | 0.9708 | 0.07441 | 0.785 |
| 1 | *CR1* | rs3818361 | 11 | *GAB2* | rs1385600 | 1.03 | 0.05009 | 0.8229 |
| 1 | *CR1* | rs3818361 | 11 | *PICALM* | rs3851179 | 0.9788 | 0.0427 | 0.8363 |
| 8 | *CLU* | rs11136000 | 11 | *GAB2* | rs4945261 | 0.979 | 0.03331 | 0.8552 |
| 8 | *CLU* | rs11136000 | 11 | *GAB2* | rs7101429 | 0.9803 | 0.02904 | 0.8647 |
| 1 | *CR1* | rs3818361 | 11 | *GAB2* | rs1007837 | 1.019 | 0.02001 | 0.8875 |
| 2 | *BIN1* | rs744373 | 19 | *APOE* | rs429358 | 1.016 | 0.01362 | 0.9071 |
| 11 | *GAB2* | rs1385600 | 11 | *PICALM* | rs3851179 | 0.9898 | 0.008679 | 0.9258 |
| 11 | *GAB2* | rs4291702 | 11 | *PICALM* | rs3851179 | 0.9898 | 0.008642 | 0.9259 |
| 2 | *BIN1* | rs744373 | 11 | *PICALM* | rs3851179 | 1.006 | 0.004565 | 0.9461 |
| 1 | *CR1* | rs3818361 | 11 | *GAB2* | rs10793294 | 0.9932 | 0.003386 | 0.9536 |
| 11 | *GAB2* | rs2373115 | 11 | *PICALM* | rs3851179 | 0.9941 | 0.002959 | 0.9566 |
| 11 | *GAB2* | rs1007837 | 11 | *PICALM* | rs3851179 | 1.003 | 0.0007777 | 0.9778 |
| 1 | *CR1* | rs3818361 | 8 | *CLU* | rs11136000 | 1.001 | 8.46E-05 | 0.9927 |
| 11 | *GAB2* | rs4945261 | 11 | *GAB2* | rs7101429 | NA | NA | NA |
