# Supplementary material for: Linking Protective GAB2 Variants, Increased Cortical GAB2 Expression and Decreased Alzheimer’s Disease Pathology
Source: PLoS One. 2013 May 28;8(5):e64802. doi: 10.1371/journal.pone.0064802 (PMC3665686; doi:10.1371/journal.pone.0064802)
Supplement: Table S3 — GAB2 mRNA levels and postmortem pathology. (DOC) [file pone.0064802.s004.doc]

**Table S3. GAB2 mRNA levels and postmortem pathology.** The diagnosis (Dx; 0=control, 1=LOAD), age-at-death (LOAD) / age-at-sampling (controls), Sex (0=male, 1=female), *APOE* E4 dose (number of E4 alleles), RNA integrity (RIN#) and ΔC_T_ values for GAB2 mRNA extracted from temporal cortex (TCX) or cerebellum (CER), neurofibrillary tangle (NFT) and senile plaque (SP) counts are shown for each sample of the post-mortem Autopsy (AUT) case-control series.

| **Subkey** | **Dx** | **Age-at-death/sampling (years)** | **Sex** | **APOE4Dose** | **RIN# CER** | **ΔC_T_ TCX** | **RIN# CER** | **ΔC_T_ CER** | **Mean no. cortical NFT** | **Mean no. cortical SP** |
| --- | --- | --- | --- | --- | --- | --- | --- | --- | --- | --- |
| 17669 | 0 | 75 | 1 | 0 | 6.5 | -0.65 | na | na | na | na |
| 17843 | 0 | 68 | 1 | 1 | 6.6 | -0.43 | 7.3 | 1.41 | 0 | 33 |
| 17532 | 0 | 77 | 0 | 0 | 5.5 | -0.42 | 6.7 | 1.27 | 0 | 0 |
| 17703 | 0 | 66 | 1 | 0 | 5.8 | -0.27 | 5.2 | 0.05 | na | na |
| 17674 | 0 | 68 | 0 | 0 | 6.3 | -0.27 | 6.7 | 1.67 | na | na |
| 17522 | 0 | 67 | 0 | 0 | 5.5 | -0.13 | 7.7 | 2.31 | 0 | 0 |
| 17650 | 0 | 77 | 1 | 1 | 6.8 | -0.08 | 8.6 | 2.25 | na | na |
| 17644 | 0 | 68 | 0 | 1 | 6.6 | -0.01 | 7 | 1.31 | na | na |
| 17754 | 0 | 72 | 0 | 0 | 7.1 | 0.04 | 5.5 | 0.67 | na | na |
| 17485 | 0 | 78 | 1 | 0 | 6.3 | 0.06 | 7.1 | 1.63 | na | na |
| 17516 | 0 | 73 | 1 | 0 | 6.4 | 0.1 | 7.1 | 1.63 | 0 | 4 |
| 17513 | 0 | 70 | 1 | 1 | 6.2 | 0.22 | 6.8 | 1.58 | 0 | 0 |
| 17658 | 0 | 79 | 1 | 0 | 6.5 | 0.28 | 6.8 | 0.81 | na | na |
| 17898 | 0 | 73 | 0 | 0 | 5.9 | 0.32 | 6.5 | 1.29 | 0 | 0 |
| 17533 | 0 | 78 | 0 | 0 | 6.1 | 0.33 | 6.9 | 1.56 | 0 | 0 |
| 17536 | 0 | 80 | 1 | 1 | 5.4 | 0.43 | 5.5 | 1.04 | 0 | 2 |
| 17666 | 0 | 61 | 0 | 0 | 7.1 | 0.51 | 6.5 | 1.39 | na | na |
| 17680 | 0 | 62 | 1 | 0 | 7.9 | 0.52 | 7.7 | 2 | na | na |
| 17732 | 0 | 68 | 0 | 0 | 6.3 | 0.59 | na | na | na | na |
| 17487 | 0 | 78 | 1 | 0 | 5.7 | 0.64 | 6.8 | 1.85 | na | na |
| 17580 | 0 | 66 | 1 | 1 | 7.8 | 0.65 | 7.7 | 1.76 | na | na |
| 17523 | 0 | 64 | 1 | 1 | 5.8 | 0.72 | 7.5 | 2.29 | 0 | 4 |
| 17715 | 0 | 72 | 0 | 0 | 6 | 0.78 | 6.9 | 1.81 | na | na |
| 17844 | 0 | 66 | 0 | 1 | 6.2 | 0.83 | 6.6 | 1.28 | 0 | 21 |
| 17525 | 0 | 65 | 0 | 0 | 5.9 | 0.83 | 7.6 | 2.09 | 0 | 0 |
| 17693 | 0 | 79 | 0 | 0 | 6.4 | 0.84 | 6.5 | 0.45 | na | na |
| 18307 | 0 | 78 | 1 | 1 | 6.4 | 0.88 | na | na | na | na |
| 17535 | 0 | 63 | 0 | 0 | 6.7 | 0.9 | 6.7 | 0.08 | 0 | 0 |
| 17571 | 0 | 76 | 1 | 1 | 8.4 | 0.9 | na | na | na | na |
| 17726 | 0 | 69 | 0 | 0 | 7 | 0.91 | 8.1 | 1.98 | na | na |
| 17517 | 0 | 79 | 0 | 0 | 6.6 | 0.95 | 7.8 | 2.61 | 0 | 0 |
| 17489 | 0 | 70 | 0 | 0 | 6.3 | 1.02 | na | na | na | na |
| 17542 | 0 | 73 | 0 | 0 | 6.9 | 1.03 | 8 | 2.63 | 0 | 0 |
| 17543 | 0 | 65 | 1 | 0 | 6.2 | 1.04 | na | na | na | na |
| 17534 | 0 | 68 | 1 | 0 | 5.6 | 1.06 | 7.4 | 2.27 | 0 | 18 |
| 17511 | 0 | 66 | 1 | 0 | 6.3 | 1.06 | na | na | na | na |
| 18105 | 0 | 80 | 1 | 0 | 6.7 | 1.07 | 7.5 | 1.82 | 0 | 10 |
| 17688 | 0 | 66 | 1 | 0 | 6.5 | 1.12 | 7.1 | 0.7 | na | na |
| 17921 | 0 | 75 | 0 | 0 | 6.7 | 1.15 | 6.6 | 1.64 | 0 | 22 |
| 17831 | 0 | 77 | 1 | 0 | 8.3 | 1.22 | 7.6 | 1.66 | 0 | 0 |
| 17828 | 0 | 71 | 0 | 1 | 6.3 | 1.23 | 5.9 | 0.94 | 0 | 11 |
| 17549 | 0 | 64 | 0 | 1 | 6.6 | 1.3 | 6.8 | 2.18 | 0 | 12 |
| 17713 | 0 | 72 | 1 | 0 | 6.3 | 1.31 | 6.7 | 1.51 | na | na |
| 17503 | 0 | 77 | 0 | 0 | 6.7 | 1.34 | 7.8 | 1.81 | na | na |
| 17740 | 0 | 70 | 1 | 0 | 6 | 1.37 | na | na | na | na |
| 17859 | 0 | 63 | 0 | 0 | 7.4 | 1.38 | 8 | 3.03 | 0 | 0 |
| 17488 | 0 | 74 | 1 | 0 | 7.4 | 1.39 | 8.6 | 2.17 | na | na |
| 17541 | 0 | 77 | 0 | 1 | 6.8 | 1.44 | 7.9 | 2.08 | 0 | 0 |
| 17574 | 0 | 79 | 0 | 0 | 6.5 | 1.48 | na | na | na | na |
| 17795 | 0 | 79 | 0 | 0 | 7 | 1.51 | 7.5 | 1.64 | 0 | 22 |
| 17819 | 0 | 68 | 0 | 0 | 5.7 | 1.6 | 7.9 | 2.37 | 0 | 0 |
| 17723 | 0 | 80 | 0 | 0 | 6.6 | 1.61 | 6.3 | 1.25 | na | na |
| 17822 | 0 | 68 | 1 | 0 | 6 | 1.62 | 7.8 | 2.52 | 0 | 0 |
| 17553 | 0 | 71 | 0 | 0 | 7.7 | 1.72 | 8.3 | 3.11 | 0 | 20 |
| 17842 | 0 | 71 | 0 | 0 | 6.3 | 1.75 | 7.6 | 2.2 | 0 | 1 |
| 18155 | 0 | 74 | 1 | 0 | 6.9 | 1.76 | 5.3 | -0.34 | 1 | 0 |
| 17509 | 0 | 70 | 0 | 1 | 7.4 | 1.77 | 7.3 | 2.04 | 0 | 0 |
| 17557 | 0 | 74 | 1 | 0 | 6.6 | 1.79 | na | na | na | na |
| 17867 | 0 | 75 | 0 | 0 | 7.6 | 1.8 | 6.7 | 1.48 | 0 | 7 |
| 17922 | 0 | 79 | 1 | 0 | 7.4 | 1.8 | 7.9 | 2.1 | 0 | 0 |
| 17839 | 0 | 66 | 1 | 0 | 7.2 | 1.94 | 5.2 | 0.36 | 0 | 8 |
| 17519 | 0 | 61 | 0 | 1 | 8.2 | 1.94 | 9 | 2.25 | 0 | 7 |
| 17730 | 0 | 78 | 1 | 0 | 7.5 | 1.94 | na | na | na | na |
| 18086 | 0 | 70 | 1 | 0 | 8.1 | 1.98 | 7 | 2.43 | 0 | 3 |
| 17591 | 0 | 78 | 0 | 1 | 8.4 | 1.99 | 8.8 | 2.86 | na | na |
| 17717 | 0 | 79 | 0 | 0 | 7.5 | 2.02 | 8.2 | 1.54 | na | na |
| 17712 | 0 | 78 | 1 | 0 | 7.5 | 2.07 | 7.2 | 1.71 | na | na |
| 17515 | 0 | 70 | 0 | 1 | 7.2 | 2.09 | 8.4 | 2.38 | 1 | 1 |
| 17744 | 0 | 68 | 0 | 0 | 7.2 | 2.14 | 7 | 1.69 | na | na |
| 17737 | 0 | 66 | 0 | 0 | 7.6 | 2.17 | na | na | na | na |
| 17906 | 0 | 65 | 0 | 0 | 8.4 | 2.2 | 7.9 | 2.35 | 0 | 0 |
| 17838 | 0 | 78 | 1 | 0 | 7.5 | 2.27 | 5.9 | 0.74 | 0 | 10 |
| 17734 | 0 | 68 | 1 | 0 | 7.9 | 2.3 | 8.7 | 2.39 | na | na |
| 17890 | 0 | 73 | 0 | 0 | 8.2 | 2.54 | na | na | na | na |
| 17529 | 0 | 78 | 0 | 0 | 8.4 | 2.56 | 8.4 | 2.46 | 0 | 9 |
| 17827 | 0 | 73 | 0 | 1 | 7.6 | 2.59 | 8.8 | 2.73 | 0 | 1 |
| 17546 | 0 | 69 | 0 | 0 | 7.3 | 2.98 | 8.1 | 2.85 | 0 | 0 |
| 17524 | 0 | 65 | 0 | 0 | 8.4 | 3.16 | 8.1 | 3.35 | 0 | 0 |
| 17623 | 0 | 78 | 1 | 1 | na | na | 5.3 | -0.7 | na | na |
| 17871 | 0 | 75 | 1 | 1 | na | na | 5.6 | -0.4 | 0 | 4 |
| 17903 | 0 | 66 | 0 | 1 | na | na | 5.1 | -0.18 | 1 | 8 |
| 17613 | 0 | 80 | 1 | 2 | na | na | 5.3 | 0.36 | na | na |
| 17864 | 0 | 70 | 0 | 0 | na | na | 5.3 | 0.4 | 0 | 0 |
| 18119 | 0 | 75 | 1 | 1 | na | na | 5.5 | 0.42 | 0 | 37 |
| 18063 | 0 | 70 | 1 | 1 | na | na | 6.2 | 0.44 | 1 | 17 |
| 18143 | 0 | 73 | 0 | 0 | na | na | 6.8 | 0.49 | na | na |
| 18091 | 0 | 80 | 0 | 0 | na | na | 5 | 0.65 | 0 | 11 |
| 17895 | 0 | 75 | 0 | 0 | na | na | 5.9 | 0.74 | 0 | 0 |
| 18006 | 0 | 71 | 0 | 1 | na | na | 6.4 | 0.82 | 0 | 2 |
| 18170 | 0 | 77 | 0 | 0 | na | na | 6.3 | 0.84 | 0 | 0 |
| 18001 | 0 | 69 | 1 | 0 | na | na | 5 | 0.91 | 0 | 0 |
| 18031 | 0 | 71 | 0 | 0 | na | na | 5.9 | 0.94 | 0 | 0 |
| 18110 | 0 | 65 | 1 | 0 | na | na | 5.5 | 0.96 | na | na |
| 18087 | 0 | 79 | 1 | 0 | na | na | 7.7 | 1.04 | 0 | 16 |
| 17879 | 0 | 74 | 0 | 0 | na | na | 5.6 | 1.12 | 0 | 0 |
| 17885 | 0 | 78 | 0 | 0 | na | na | 7.6 | 1.19 | 0 | 2 |
| 17992 | 0 | 76 | 1 | 0 | na | na | 5 | 1.19 | 0 | 14 |
| 17829 | 0 | 70 | 0 | 0 | na | na | 6.5 | 1.21 | 0 | 0 |
| 18113 | 0 | 76 | 0 | 0 | na | na | 5.7 | 1.21 | 0 | 0 |
| 18000 | 0 | 70 | 1 | 0 | na | na | 7.1 | 1.22 | 0 | 0 |
| 18045 | 0 | 70 | 0 | 0 | na | na | 6.5 | 1.23 | 0 | 26 |
| 17953 | 0 | 77 | 0 | 0 | na | na | 6.1 | 1.26 | 0 | 7 |
| 17918 | 0 | 74 | 0 | 0 | na | na | 6.7 | 1.28 | 0 | 0 |
| 18285 | 0 | 78 | 1 | 1 | na | na | 8.1 | 1.28 | na | na |
| 17609 | 0 | 73 | 0 | 0 | na | na | 6.1 | 1.29 | na | na |
| 17852 | 0 | 78 | 0 | 0 | na | na | 5.5 | 1.29 | 0 | 0 |
| 17826 | 0 | 69 | 1 | 0 | na | na | 6.7 | 1.31 | 0 | 5 |
| 17837 | 0 | 71 | 0 | 0 | na | na | 6.4 | 1.33 | 0 | 0 |
| 18060 | 0 | 77 | 1 | 1 | na | na | 6.4 | 1.36 | 0 | 39 |
| 17902 | 0 | 79 | 0 | 1 | na | na | 6.7 | 1.39 | 0 | 15 |
| 17968 | 0 | 73 | 0 | 1 | na | na | 6.6 | 1.42 | 0 | 1 |
| 17585 | 0 | 73 | 0 | 0 | na | na | 7.1 | 1.45 | na | na |
| 17886 | 0 | 75 | 1 | 0 | na | na | 7.5 | 1.59 | 0 | 0 |
| 17954 | 0 | 68 | 0 | 0 | na | na | 7.1 | 1.59 | 0 | 1 |
| 17823 | 0 | 73 | 0 | 0 | na | na | 7 | 1.6 | 0 | 0 |
| 17655 | 0 | 70 | 0 | 1 | na | na | 7.3 | 1.61 | na | na |
| 18073 | 0 | 75 | 0 | 2 | na | na | 7.1 | 1.61 | 0 | 31 |
| 18132 | 0 | 69 | 0 | 0 | na | na | 6.8 | 1.64 | na | na |
| 17969 | 0 | 73 | 1 | 0 | na | na | 6.7 | 1.65 | 0 | 8 |
| 17629 | 0 | 63 | 1 | 1 | na | na | 6.7 | 1.66 | 0 | 36 |
| 17961 | 0 | 79 | 1 | 0 | na | na | 7.7 | 1.66 | 0 | 8 |
| 17877 | 0 | 70 | 1 | 0 | na | na | 7 | 1.68 | 0 | 0 |
| 17865 | 0 | 74 | 0 | 0 | na | na | 6.9 | 1.7 | 0 | 0 |
| 17920 | 0 | 66 | 1 | 0 | na | na | 7 | 1.7 | 0 | 0 |
| 18025 | 0 | 78 | 0 | 0 | na | na | 6.4 | 1.72 | 0 | 8 |
| 18023 | 0 | 73 | 1 | 0 | na | na | 6.4 | 1.74 | 0 | 0 |
| 17889 | 0 | 71 | 1 | 0 | na | na | 6.5 | 1.79 | 0 | 0 |
| 18145 | 0 | 76 | 0 | 0 | na | na | 7.9 | 1.8 | na | na |
| 17816 | 0 | 65 | 0 | 1 | na | na | 6.4 | 1.81 | 0 | 0 |
| 17814 | 0 | 71 | 0 | 0 | na | na | 7.7 | 1.82 | na | na |
| 17857 | 0 | 77 | 0 | 0 | na | na | 6.3 | 1.82 | 0 | 3 |
| 18064 | 0 | 75 | 1 | 0 | na | na | 7.9 | 1.82 | 0 | 24 |
| 17667 | 0 | 70 | 0 | 0 | na | na | 6.7 | 1.88 | na | na |
| 17861 | 0 | 62 | 0 | 0 | na | na | 7.5 | 1.88 | 0 | 0 |
| 18134 | 0 | 70 | 0 | 0 | na | na | 7.3 | 1.92 | na | na |
| 17625 | 0 | 78 | 0 | 1 | na | na | 7.2 | 1.93 | na | na |
| 18056 | 0 | 75 | 0 | 0 | na | na | 7.5 | 1.95 | 0 | 43 |
| 17914 | 0 | 65 | 0 | 0 | na | na | 7.6 | 1.98 | 0 | 3 |
| 18026 | 0 | 77 | 0 | 0 | na | na | 5.9 | 2.07 | 2 | 0 |
| 18305 | 0 | 77 | 1 | 1 | na | na | 7.9 | 2.08 | na | na |
| 17971 | 0 | 74 | 1 | 0 | na | na | 8.3 | 2.09 | 0 | 16 |
| 18033 | 0 | 70 | 0 | 0 | na | na | 7.2 | 2.09 | 0 | 1 |
| 17661 | 0 | 68 | 0 | 0 | na | na | 8.3 | 2.12 | na | na |
| 17736 | 0 | 65 | 0 | 1 | na | na | 8.1 | 2.12 | na | na |
| 18107 | 0 | 64 | 0 | 0 | na | na | 7.8 | 2.13 | 0 | 0 |
| 17854 | 0 | 69 | 0 | 0 | na | na | 7.3 | 2.15 | 0 | 1 |
| 17907 | 0 | 70 | 0 | 1 | na | na | 6.9 | 2.15 | 0 | 1 |
| 17988 | 0 | 79 | 1 | 0 | na | na | 7.9 | 2.15 | 8 | 0 |
| 18112 | 0 | 63 | 0 | 0 | na | na | 7.8 | 2.17 | 0 | 0 |
| 17670 | 0 | 74 | 0 | 1 | na | na | 8 | 2.19 | na | na |
| 17893 | 0 | 77 | 1 | 0 | na | na | 8.3 | 2.2 | 0 | 0 |
| 18085 | 0 | 78 | 1 | 0 | na | na | 7.4 | 2.21 | 0 | 11 |
| 17478 | 0 | 79 | 0 | 0 | na | na | 7.2 | 2.23 | 0 | 34 |
| 17873 | 0 | 62 | 1 | 0 | na | na | 7.9 | 2.24 | 0 | 0 |
| 18084 | 0 | 69 | 0 | 0 | na | na | 8 | 2.24 | 0 | 13 |
| 18414 | 0 | 78 | 0 | 1 | na | na | 7.7 | 2.25 | na | na |
| 17821 | 0 | 68 | 0 | 0 | na | na | 7.9 | 2.26 | 0 | 5 |
| 18082 | 0 | 69 | 0 | 0 | na | na | 7 | 2.28 | 0 | 1 |
| 17657 | 0 | 68 | 0 | 0 | na | na | 8.4 | 2.29 | na | na |
| 18059 | 0 | 66 | 1 | 1 | na | na | 8.6 | 2.29 | 0 | 24 |
| 17860 | 0 | 62 | 0 | 0 | na | na | 7.7 | 2.31 | 1 | 0 |
| 18009 | 0 | 72 | 1 | 0 | na | na | 9 | 2.35 | 0 | 0 |
| 17880 | 0 | 79 | 1 | 0 | na | na | 8.2 | 2.37 | 1 | 1 |
| 18127 | 0 | 78 | 0 | 1 | na | na | 8.3 | 2.38 | na | na |
| 17752 | 0 | 68 | 0 | 0 | na | na | 7.2 | 2.41 | na | na |
| 17866 | 0 | 75 | 1 | 0 | na | na | 7.4 | 2.45 | 0 | 0 |
| 18236 | 0 | 79 | 1 | 2 | na | na | 8.7 | 2.45 | na | na |
| 17995 | 0 | 67 | 0 | 0 | na | na | 8.2 | 2.46 | 0 | 0 |
| 17714 | 0 | 79 | 0 | 1 | na | na | 7.7 | 2.48 | na | na |
| 17913 | 0 | 73 | 0 | 0 | na | na | 8 | 2.5 | 0 | 0 |
| 17679 | 0 | 74 | 0 | 1 | na | na | 7.5 | 2.51 | na | na |
| 18097 | 0 | 78 | 1 | 0 | na | na | 7.8 | 2.56 | 0 | 9 |
| 17892 | 0 | 74 | 0 | 0 | na | na | 8.1 | 2.57 | 0 | 4 |
| 18034 | 0 | 70 | 0 | 0 | na | na | 7.6 | 2.59 | 1 | 0 |
| 18294 | 0 | 76 | 1 | 0 | na | na | 7.8 | 2.63 | na | na |
| 17671 | 0 | 67 | 0 | 0 | na | na | 7.9 | 2.74 | na | na |
| 18083 | 0 | 72 | 0 | 0 | na | na | 8.5 | 2.79 | 0 | 0 |
| 17897 | 0 | 74 | 0 | 0 | na | na | 7.8 | 2.8 | 0 | 2 |
| 18274 | 0 | 79 | 0 | 1 | na | na | 7.8 | 2.9 | na | na |
| 17960 | 0 | 70 | 0 | 0 | na | na | 8 | 2.91 | 0 | 2 |
| 17956 | 0 | 67 | 0 | 0 | na | na | 8.6 | 2.94 | 0 | 0 |
| 17894 | 0 | 65 | 1 | 0 | na | na | 7.8 | 2.97 | 0 | 0 |
| 18058 | 0 | 75 | 0 | 1 | na | na | 8.3 | 2.99 | 0 | 40 |
| 18124 | 0 | 79 | 0 | 1 | na | na | 8.1 | 3.02 | na | na |
| 17080 | 1 | 73 | 1 | 0 | 5.6 | -1.82 | 6.1 | 1 | 21 | 44 |
| 16978 | 1 | 77 | 1 | 2 | 5.9 | -1.09 | 6.2 | 0.86 | 17 | 50 |
| 17400 | 1 | 72 | 1 | 1 | 6.2 | -0.88 | 7.4 | 2.66 | 16 | 48 |
| 16914 | 1 | 68 | 1 | 2 | 6.4 | -0.65 | 7.9 | 2.05 | 5 | 36 |
| 17217 | 1 | 73 | 1 | 2 | 6.2 | -0.54 | 6.1 | 1.18 | na | na |
| 16860 | 1 | 65 | 1 | 0 | 5.8 | -0.5 | na | na | na | na |
| 17362 | 1 | 61 | 1 | 2 | 5.9 | -0.3 | 7.2 | 1.85 | na | na |
| 17025 | 1 | 75 | 1 | 0 | 6.5 | -0.27 | 8.1 | 1.63 | 16 | 50 |
| 17142 | 1 | 70 | 1 | 0 | 5.6 | -0.24 | 7.3 | 1.39 | 6 | 48 |
| 17121 | 1 | 79 | 1 | 1 | 6.1 | -0.12 | 7.9 | 1.26 | 9 | 41 |
| 17281 | 1 | 78 | 1 | 1 | 6.3 | -0.1 | 9.2 | 2.28 | na | na |
| 17051 | 1 | 72 | 1 | 0 | 6.2 | -0.04 | 7.7 | 2.08 | 12 | 34 |
| 17440 | 1 | 79 | 0 | 1 | 6.3 | 0.06 | 6.6 | 1.72 | 6 | 50 |
| 17332 | 1 | 61 | 1 | 1 | 7.2 | 0.09 | 6.8 | -0.2 | 20 | 50 |
| 17099 | 1 | 76 | 0 | 1 | 6.1 | 0.12 | 7 | 1.15 | 7 | 46 |
| 17165 | 1 | 74 | 1 | 0 | 6.3 | 0.12 | 7.4 | 1.71 | 10 | 47 |
| 17343 | 1 | 77 | 1 | 0 | 6.7 | 0.16 | 7.2 | 2.11 | na | na |
| 17176 | 1 | 80 | 1 | 0 | 6.5 | 0.22 | 7.9 | 2.59 | 11 | 43 |
| 16942 | 1 | 74 | 0 | 0 | 7.8 | 0.24 | 8.2 | 2.25 | 9 | 37 |
| 17092 | 1 | 74 | 0 | 0 | 6.2 | 0.26 | 7.5 | 2.24 | 14 | 38 |
| 17475 | 1 | 80 | 1 | 1 | 6.3 | 0.29 | 7.4 | 1.77 | 17 | 46 |
| 17023 | 1 | 71 | 1 | 0 | 6.7 | 0.3 | 8.1 | 3.27 | 12 | 39 |
| 17095 | 1 | 62 | 1 | 0 | 5.8 | 0.33 | 6.2 | 0.4 | 12 | 44 |
| 17375 | 1 | 80 | 0 | 1 | 6.2 | 0.33 | 6.7 | 1.56 | na | na |
| 16905 | 1 | 75 | 1 | 1 | 6.5 | 0.35 | 6.6 | 2.15 | 24 | 47 |
| 16931 | 1 | 80 | 1 | 0 | 6.6 | 0.35 | 8.4 | 2.55 | 7 | 45 |
| 17327 | 1 | 78 | 0 | 0 | 6.7 | 0.37 | 6.8 | 1.59 | 17 | 38 |
| 17212 | 1 | 72 | 0 | 1 | 6.6 | 0.46 | 8 | 2.02 | na | na |
| 16991 | 1 | 66 | 0 | 1 | 6.7 | 0.53 | 7.3 | 1.94 | 20 | 43 |
| 17467 | 1 | 79 | 0 | 1 | 6.6 | 0.56 | 7.9 | 2.36 | 1 | 39 |
| 17265 | 1 | 80 | 0 | 0 | 6.1 | 0.56 | 7.3 | 2.63 | na | na |
| 17078 | 1 | 77 | 1 | 1 | 6.7 | 0.66 | 7.6 | 2.64 | 8 | 36 |
| 17143 | 1 | 75 | 0 | 2 | 6.4 | 0.67 | 6.8 | 0.34 | 11 | 32 |
| 17378 | 1 | 63 | 0 | 1 | 6.3 | 0.67 | 7.2 | 1.44 | na | na |
| 17002 | 1 | 68 | 0 | 1 | 6.8 | 0.69 | 8.3 | 2.05 | 8 | 44 |
| 17036 | 1 | 75 | 0 | 0 | 7.9 | 0.7 | 7.6 | 1.31 | 3 | 38 |
| 17086 | 1 | 77 | 0 | 1 | 6.7 | 0.71 | 8 | 1.64 | 15 | 50 |
| 17088 | 1 | 67 | 1 | 1 | 5.7 | 0.76 | 5.9 | 0.46 | 16 | 45 |
| 17406 | 1 | 80 | 0 | 1 | 7 | 0.79 | 7.7 | 2.42 | 1 | 34 |
| 17360 | 1 | 75 | 0 | 1 | 6.1 | 0.8 | 6 | 1.51 | na | na |
| 16936 | 1 | 74 | 1 | 1 | 6.7 | 0.8 | 6.9 | 2.17 | 20 | 49 |
| 17104 | 1 | 76 | 0 | 0 | 6.3 | 0.8 | 7.4 | 2.3 | 16 | 49 |
| 17474 | 1 | 80 | 1 | 0 | 6.7 | 0.91 | 7.1 | 1.24 | 11 | 36 |
| 17140 | 1 | 80 | 0 | 1 | 6.3 | 0.96 | 7.3 | 2.04 | 10 | 40 |
| 17100 | 1 | 80 | 0 | 1 | 6.7 | 0.97 | 7.6 | 1.44 | 13 | 42 |
| 16911 | 1 | 76 | 0 | 0 | 6.5 | 1 | 8.2 | 2.4 | 15 | 46 |
| 17083 | 1 | 80 | 0 | 1 | 6.2 | 1.06 | 8.8 | 2.13 | 7 | 43 |
| 16948 | 1 | 78 | 1 | 0 | 7.9 | 1.1 | 7.9 | 1.91 | 8 | 38 |
| 16924 | 1 | 78 | 0 | 0 | 6.8 | 1.13 | 7.7 | 1.09 | 14 | 44 |
| 17377 | 1 | 79 | 1 | 1 | 6.9 | 1.13 | 8.2 | 2.18 | na | na |
| 16929 | 1 | 79 | 1 | 0 | 6.6 | 1.15 | 7.4 | 1.57 | na | na |
| 17029 | 1 | 65 | 0 | 0 | 6.7 | 1.17 | 5.6 | 0.84 | na | na |
| 17150 | 1 | 73 | 0 | 1 | 7.1 | 1.18 | 7.8 | 2 | 6 | 34 |
| 16989 | 1 | 73 | 1 | 2 | 7.9 | 1.19 | 8.7 | 2.15 | 11 | 32 |
| 16930 | 1 | 62 | 1 | 1 | 8.9 | 1.24 | 9.4 | 2.43 | 13 | 44 |
| 17009 | 1 | 79 | 0 | 1 | 6.8 | 1.26 | 7.1 | 1.8 | 2 | 41 |
| 17430 | 1 | 77 | 0 | 0 | 7.2 | 1.3 | 7.9 | 2.08 | 6 | 45 |
| 17459 | 1 | 80 | 0 | 1 | 6.8 | 1.31 | 6.6 | 2.08 | 5 | 45 |
| 17249 | 1 | 74 | 0 | 0 | 7.5 | 1.32 | 8.2 | 2.31 | na | na |
| 16940 | 1 | 66 | 0 | 1 | 8.1 | 1.32 | 8.6 | 2.53 | 12 | 50 |
| 16941 | 1 | 70 | 0 | 1 | 6.9 | 1.42 | 8 | 2.5 | 24 | 47 |
| 17135 | 1 | 79 | 0 | 1 | 7.9 | 1.43 | 8.7 | 2.58 | 11 | 38 |
| 17153 | 1 | 66 | 0 | 2 | 7.1 | 1.44 | 7.7 | 0.68 | na | na |
| 17252 | 1 | 80 | 0 | 0 | 7.6 | 1.48 | 8.5 | 2.72 | na | na |
| 17244 | 1 | 73 | 1 | 2 | 6.2 | 1.5 | 8 | 1.97 | na | na |
| 17133 | 1 | 78 | 1 | 0 | 7.4 | 1.52 | 7.1 | 1.84 | 3 | 37 |
| 17171 | 1 | 80 | 0 | 0 | 7 | 1.54 | 5.5 | 2 | 5 | 40 |
| 17274 | 1 | 78 | 1 | 1 | 6.2 | 1.55 | 6.3 | 0.92 | na | na |
| 17209 | 1 | 73 | 1 | 1 | 6.7 | 1.63 | 7.6 | 2.04 | 2 | 42 |
| 17043 | 1 | 63 | 1 | 0 | 7.4 | 1.63 | 9 | 2.06 | 16 | 43 |
| 17040 | 1 | 71 | 0 | 1 | 6.7 | 1.69 | 7.3 | 2.04 | 5 | 32 |
| 17073 | 1 | 65 | 0 | 1 | 7 | 1.71 | 7.9 | 1.63 | 11 | 50 |
| 17251 | 1 | 72 | 0 | 1 | 8.3 | 1.71 | 8.6 | 2.93 | na | na |
| 17066 | 1 | 78 | 0 | 2 | 9 | 1.9 | 7.9 | 1.74 | 9 | 45 |
| 17035 | 1 | 74 | 0 | 1 | 7.8 | 1.97 | 7.6 | 2.32 | 3 | 34 |
| 17325 | 1 | 80 | 1 | 1 | 7.4 | 2.02 | 8.4 | 1.92 | na | na |
| 17351 | 1 | 70 | 1 | 1 | 7.3 | 2.04 | 8 | 1.81 | na | na |
| 17385 | 1 | 69 | 0 | 1 | 6.9 | 2.05 | 8.4 | 2.12 | na | na |
| 17286 | 1 | 79 | 0 | 1 | 7.9 | 2.05 | 8.2 | 2.25 | na | na |
| 17063 | 1 | 78 | 1 | 0 | 7.3 | 2.06 | 8.9 | 2.53 | 16 | 45 |
| 17003 | 1 | 77 | 1 | 0 | 6.7 | 2.16 | 8.4 | 2.42 | 13 | 39 |
| 17242 | 1 | 73 | 0 | 2 | 8.1 | 2.2 | 7.6 | 2.12 | na | na |
| 17085 | 1 | 77 | 0 | 2 | 7.3 | 2.23 | 8.4 | 2.01 | 17 | 46 |
| 17237 | 1 | 75 | 1 | 1 | 8.1 | 2.28 | 6.9 | 1.97 | na | na |
| 17273 | 1 | 77 | 0 | 0 | 9 | 2.54 | 7.5 | 1.75 | na | na |
| 16893 | 1 | 73 | 1 | 1 | na | na | 5.4 | -1.11 | 17 | 45 |
| 17457 | 1 | 78 | 0 | 1 | na | na | 5.5 | -0.82 | 4 | 37 |
| 17382 | 1 | 65 | 1 | 0 | na | na | 5.1 | -0.64 | na | na |
| 17379 | 1 | 79 | 1 | 2 | na | na | 5.6 | -0.05 | na | na |
| 16966 | 1 | 75 | 0 | 0 | na | na | 6.3 | 0.02 | 9 | 47 |
| 16970 | 1 | 80 | 0 | 0 | na | na | 5.7 | 0.2 | 13 | 46 |
| 17032 | 1 | 61 | 0 | 0 | na | na | 6 | 0.23 | 18 | 44 |
| 17071 | 1 | 73 | 1 | 2 | na | na | 6.9 | 0.32 | 24 | 50 |
| 17284 | 1 | 75 | 1 | 1 | na | na | 5.1 | 0.38 | na | na |
| 17425 | 1 | 79 | 0 | 1 | na | na | 5 | 0.4 | 8 | 37 |
| 17082 | 1 | 66 | 0 | 1 | na | na | 5.4 | 0.42 | 16 | 46 |
| 17118 | 1 | 76 | 0 | 0 | na | na | 5.8 | 0.43 | 17 | 39 |
| 17094 | 1 | 74 | 1 | 1 | na | na | 5.2 | 0.48 | 16 | 45 |
| 16962 | 1 | 68 | 1 | 0 | na | na | 5.1 | 0.51 | 34 | 50 |
| 17069 | 1 | 77 | 0 | 0 | na | na | 5.4 | 0.6 | 15 | 45 |
| 17134 | 1 | 76 | 1 | 0 | na | na | 5.6 | 0.7 | 10 | 44 |
| 17246 | 1 | 79 | 1 | 1 | na | na | 6.3 | 0.77 | na | na |
| 17438 | 1 | 77 | 0 | 1 | na | na | 5.9 | 0.81 | 9 | 45 |
| 16968 | 1 | 70 | 1 | 0 | na | na | 6.2 | 0.92 | 19 | 45 |
| 17059 | 1 | 80 | 1 | 1 | na | na | 6.4 | 0.92 | 4 | 44 |
| 17410 | 1 | 67 | 1 | 1 | na | na | 5.5 | 1 | 13 | 45 |
| 17240 | 1 | 74 | 1 | 1 | na | na | 6.4 | 1.01 | na | na |
| 17026 | 1 | 68 | 1 | 0 | na | na | 7 | 1.03 | 8 | 44 |
| 17387 | 1 | 74 | 1 | 0 | na | na | 6 | 1.05 | na | na |
| 17008 | 1 | 73 | 1 | 0 | na | na | 7 | 1.07 | 6 | 45 |
| 16908 | 1 | 79 | 0 | 1 | na | na | 5.7 | 1.1 | 5 | 41 |
| 17279 | 1 | 73 | 0 | 1 | na | na | 6.3 | 1.1 | na | na |
| 16904 | 1 | 61 | 1 | 0 | na | na | 7.4 | 1.13 | 10 | 41 |
| 17091 | 1 | 73 | 1 | 1 | na | na | 7 | 1.16 | 4 | 39 |
| 17388 | 1 | 68 | 0 | 0 | na | na | 5.6 | 1.2 | na | na |
| 17390 | 1 | 72 | 0 | 0 | na | na | 6.1 | 1.2 | na | na |
| 17258 | 1 | 77 | 0 | 1 | na | na | 7.2 | 1.24 | na | na |
| 17461 | 1 | 79 | 1 | 0 | na | na | 5.7 | 1.25 | 8 | 32 |
| 17149 | 1 | 79 | 1 | 1 | na | na | 5.9 | 1.26 | 9 | 47 |
| 17316 | 1 | 70 | 0 | 1 | na | na | 6.5 | 1.28 | 13 | 42 |
| 17225 | 1 | 75 | 1 | 0 | na | na | 6 | 1.29 | na | na |
| 16983 | 1 | 68 | 0 | 1 | na | na | 6.3 | 1.33 | 7 | 15 |
| 17227 | 1 | 77 | 0 | 1 | na | na | 7 | 1.38 | na | na |
| 16906 | 1 | 76 | 1 | 1 | na | na | 6.3 | 1.41 | 13 | 49 |
| 17007 | 1 | 68 | 1 | 1 | na | na | 6.2 | 1.41 | na | na |
| 17303 | 1 | 75 | 0 | 1 | na | na | 6.6 | 1.43 | 2 | 43 |
| 17226 | 1 | 70 | 0 | 0 | na | na | 6.9 | 1.47 | na | na |
| 17442 | 1 | 80 | 1 | 0 | na | na | 6.7 | 1.49 | 8 | 46 |
| 17079 | 1 | 67 | 0 | 2 | na | na | 6.6 | 1.52 | 22 | 50 |
| 16938 | 1 | 80 | 0 | 1 | na | na | 8.6 | 1.55 | 7 | 32 |
| 17464 | 1 | 80 | 1 | 1 | na | na | 6.7 | 1.59 | 23 | 45 |
| 16949 | 1 | 77 | 1 | 0 | na | na | 6.1 | 1.6 | 4 | 30 |
| 17114 | 1 | 62 | 0 | 0 | na | na | 7.3 | 1.6 | 11 | 45 |
| 17380 | 1 | 70 | 0 | 1 | na | na | 6.3 | 1.62 | na | na |
| 17144 | 1 | 80 | 1 | 1 | na | na | 7.6 | 1.64 | 14 | 48 |
| 17228 | 1 | 62 | 1 | 2 | na | na | 6.7 | 1.65 | na | na |
| 17230 | 1 | 70 | 1 | 0 | na | na | 7.6 | 1.68 | na | na |
| 16920 | 1 | 71 | 0 | 2 | na | na | 7 | 1.69 | na | na |
| 17112 | 1 | 79 | 1 | 1 | na | na | 7.4 | 1.71 | 10 | 40 |
| 16971 | 1 | 74 | 1 | 2 | na | na | 7.5 | 1.72 | 22 | 49 |
| 17270 | 1 | 70 | 1 | 0 | na | na | 7.1 | 1.73 | na | na |
| 17415 | 1 | 77 | 1 | 1 | na | na | 6.5 | 1.73 | 9 | 37 |
| 17101 | 1 | 69 | 0 | 0 | na | na | 6.6 | 1.75 | 10 | 49 |
| 17239 | 1 | 80 | 0 | 0 | na | na | 7.1 | 1.78 | na | na |
| 17211 | 1 | 70 | 0 | 2 | na | na | 7.1 | 1.82 | na | na |
| 16995 | 1 | 79 | 0 | 1 | na | na | 6.6 | 1.85 | 8 | 39 |
| 17183 | 1 | 62 | 1 | 2 | na | na | 6.3 | 1.85 | 7 | 50 |
| 17216 | 1 | 74 | 1 | 1 | na | na | 8.8 | 1.86 | na | na |
| 17229 | 1 | 74 | 0 | 1 | na | na | 7.5 | 1.87 | na | na |
| 17320 | 1 | 69 | 1 | 0 | na | na | 7.6 | 1.88 | 12 | 49 |
| 17072 | 1 | 69 | 1 | 2 | na | na | 6.9 | 1.9 | 17 | 49 |
| 17253 | 1 | 80 | 1 | 1 | na | na | 7.9 | 1.91 | na | na |
| 16939 | 1 | 75 | 1 | 0 | na | na | 7.2 | 1.93 | 12 | 50 |
| 17141 | 1 | 76 | 0 | 2 | na | na | 7.3 | 1.94 | 11 | 44 |
| 17103 | 1 | 70 | 1 | 0 | na | na | 7 | 1.95 | 17 | 45 |
| 16927 | 1 | 63 | 0 | 1 | na | na | 6.8 | 1.96 | na | na |
| 17000 | 1 | 74 | 1 | 2 | na | na | 7.5 | 1.97 | 18 | 50 |
| 17013 | 1 | 78 | 0 | 0 | na | na | 7.4 | 1.97 | 25 | 43 |
| 17200 | 1 | 69 | 1 | 1 | na | na | 6.5 | 1.98 | na | na |
| 17530 | 1 | 79 | 0 | 1 | na | na | 7.5 | 2 | 5 | 42 |
| 17411 | 1 | 79 | 1 | 1 | na | na | 7 | 2.01 | 13 | 46 |
| 17137 | 1 | 73 | 0 | 0 | na | na | 9.4 | 2.03 | 12 | 20 |
| 16979 | 1 | 79 | 0 | 0 | na | na | 7.2 | 2.07 | 4 | 40 |
| 17222 | 1 | 70 | 1 | 1 | na | na | 7.2 | 2.07 | na | na |
| 17067 | 1 | 77 | 1 | 2 | na | na | 7.2 | 2.22 | 27 | 49 |
| 17448 | 1 | 76 | 1 | 1 | na | na | 7.9 | 2.23 | 15 | 43 |
| 17269 | 1 | 78 | 0 | 1 | na | na | 8 | 2.24 | na | na |
| 16976 | 1 | 72 | 1 | 1 | na | na | 7.2 | 2.25 | 13 | 41 |
| 17292 | 1 | 78 | 0 | 2 | na | na | 7.7 | 2.26 | na | na |
| 17354 | 1 | 71 | 1 | 1 | na | na | 8 | 2.26 | na | na |
| 16928 | 1 | 79 | 1 | 2 | na | na | 7.7 | 2.29 | 7 | 50 |
| 16915 | 1 | 80 | 1 | 0 | na | na | 7.3 | 2.3 | 12 | 41 |
| 16986 | 1 | 79 | 0 | 1 | na | na | 8.2 | 2.3 | 7 | 44 |
| 17161 | 1 | 80 | 1 | 0 | na | na | 8.1 | 2.3 | 13 | 43 |
| 17021 | 1 | 61 | 0 | 1 | na | na | 8.2 | 2.34 | 14 | 50 |
| 17257 | 1 | 75 | 1 | 1 | na | na | 8.2 | 2.35 | na | na |
| 17297 | 1 | 76 | 0 | 1 | na | na | 8.1 | 2.37 | 4 | 30 |
| 17019 | 1 | 69 | 0 | 2 | na | na | 7.3 | 2.5 | 14 | 43 |
| 17346 | 1 | 74 | 1 | 1 | na | na | 8.5 | 2.5 | na | na |
| 16965 | 1 | 61 | 0 | 0 | na | na | 7.3 | 2.55 | 12 | 48 |
| 17931 | 1 | 74 | 0 | 0 | na | na | 8.2 | 2.56 | 6 | 49 |
| 16879 | 1 | 73 | 0 | 0 | na | na | 6.1 | 2.6 | 11 | 45 |
| 17231 | 1 | 71 | 1 | 1 | na | na | 8.1 | 2.61 | na | na |
| 16889 | 1 | 64 | 0 | 1 | na | na | 8.9 | 2.67 | 20 | 50 |
| 17403 | 1 | 77 | 0 | 1 | na | na | 8.8 | 2.7 | 5 | 36 |
| 16954 | 1 | 67 | 1 | 0 | na | na | 7 | 3.18 | 18 | 48 |
